# *Piezo1*-expressing vocal fold epithelia modulate remodeling via effects on self-renewal and cytokeratin differentiation

**DOI:** 10.1007/s00018-022-04622-6

**Published:** 2022-11-14

**Authors:** Alexander G. Foote, Vlasta Lungova, Susan L. Thibeault

**Affiliations:** Division of Otolaryngology-Head and Neck Surgery, Department of Surgery, University of Wisconsin-Madison, Wisconsin, USA

**Keywords:** Vocal folds, Wound healing, Regeneration, Epithelial mechanoreceptors, Mechanobiology

## Abstract

Mechanoreceptors are implicated as functional afferents within mucosa of the airways and the recent discovery of mechanosensitive channels *Piezo1* and *Piezo2* has proved essential for cells of various mechanically sensitive tissues. However, the role for *Piezo1/2* in vocal fold (VF) mucosal epithelia, a cell that withstands excessive biomechanical insult, remains unknown. The purpose of this study was to test the hypothesis that *Piezo1* is required for VF mucosal repair pathways of epithelial cell injury. Utilizing a *sonic hedgehog* (*shh*) Cre line for epithelial-specific ablation of *Piezo1/2* mechanoreceptors, we investigated 6wk adult VF mucosa following naphthalene exposure for repair strategies at 1, 3, 7 and 14 days post-injury (dpi). PIEZO1 localized to differentiated apical epithelia and was paramount for epithelial remodeling events. Injury to wildtype epithelium was most appreciated at 3 dpi. *Shh*^*cre/*+^; *Piezo1*^*loxP/loxP*^, *Piezo2 *^*loxP/*+^ mutant epithelium exhibited severe cell/nuclear defects compared to injured controls. Conditional ablation of *Piezo1* and/or *Piezo2* to uninjured VF epithelium did not result in abnormal phenotypes across P0, P15 and 6wk postnatal stages compared to heterozygote and control tissue. Results demonstrate a role for *Piezo1*-expressing VF epithelia in regulating self-renewal via effects on *p63* transcription and YAP subcellular translocation—altering cytokeratin differentiation.

**Supplementary Information:**

The online version contains supplementary material available at 10.1007/s00018-022-04622-6.

## Introduction

The larynx, situated along the airway, is richly innervated with nerves [1, 2]. Diversity of physiological inputs from peripheral afferents in mucosa [3–12] serves to elicit characteristic reflexes to guard and/or clear the airways [13, 14]. Free margins of the vocal fold (VF), covered by stratified squamous epithelium (SSE), sustain extensive physiological stress during the biomechanical acts of voicing and cough [15–21]. Under homeostatic conditions, cells are largely quiescent, exhibiting low rates of proliferation and cellular turnover. However, when significant disruption occurs in response to injury and insult, rapid VF epithelium regeneration is critical for long-term tissue survival and maintenance. Analogous to other tissues, acute VF mucosal injury initiates a tissue repair process comprising of overlapping wound healing cellular phases involving: (1) inflammation, (2) proliferation and re-epithelialization and (3) ECM synthesis and remodeling [22–25]. VF mucosal epithelial cells, not only offer self-renewal capacity but, provide first-line defense against physical and chemical insult from the laryngeal lumen [17, 26, 27], requiring an intact protective barrier [15].

As few other organs and/or tissues withstand such shearing and frictional forces during motion [28], VF epithelial cells represent a unique cell type to study mechanotransduction. In 2010, a new group of mammalian mechanically activated ion channels, *Piezo1* and *Piezo2* were discovered [29], prompting a surge of research into their functional roles in cells. Since their discovery, *Piezo1* channels have been found to be inherently mechanosensitive [30], predominantly expressed in nonexcitable cell types, and shown to regulate the life cycle of epithelial cells [31–33]. In contrast, *Piezo2* channels have been found primarily expressed in sensory neurons [29, 34–39], regulating critical roles in sensory processes such as gentle touch sensation [40], proprioception [41] and mechanical nociception [42].

Given challenging biomechanical vibration within the larynx and subjected to VF tissue [18], it is not surprising that rapidly adapting mechanoreceptors have been implicated as functional afferents in laryngeal mucosa [10]. However, current research has relied on observational studies (e.g. punctate/air pulse mechanical stimulation) [43–45] and electrophysiological responses in vagal activity [46], with little underlying biological evidence at the mucosa–airway interface. Furthermore, PIEZO1/2 localization to laryngeal epithelial cells has not been explored, with initial evidence suggesting airway threats may act upon upstream epithelial sentinel cells rather than directly on neurons [13, 47].

The purpose of this study was to test the hypothesis that *Piezo1*-expressing ion channels to VF epithelia are essential for differentiation of SSE during postnatal life, and function to modulate acute remodeling following injury. We adopted a relatively established murine model for lower airway epithelial injury using chemical naphthalene [48–54]; not previously tested in the upper airway. Utilizing a *sonic hedgehog* (*shh*) Cre line for epithelial-specific ablation of *Piezo1/2* mechanoreceptors, we investigated 6wk adult VF mucosa for repair strategies at 1, 3-, 7 and 14 days post-injury (dpi). We found that naphthalene facilitates VF mucosal injury with a noninvasive, reproducible and epithelial-targeted approach—most appreciated at 3 dpi in both wildtype and mutant colonies. Results demonstrated the role for *Piezo1* in modulating self-renewal via effects on *p63* transcription and YAP protein subcellular translocation. We noted irregular cytokeratin differentiation for downstream stratification events, most notably, ectopic K17 expression, with unique changes to the subglottis at the squamociliary junction. Regenerating epithelium was restored by 7 dpi for all genotypes and treatment conditions, albeit, by 14 dpi ZO1-marked tight junctions to apical cells were impaired for both uninjured and injured *Piezo1* ablated epithelium, suggesting continued barrier compromise. Findings from this investigation improve our understanding of acute VF wound healing in the context of *Piezo1* epithelial function and form the basis for an in vivo methodology to study VF responses to naphthalene injury.

## Materials and methods

### Mouse models and tissue harvesting

*Shh*gfpcre (JAX stock #05622), *Piezo1*flox (JAX stock #029213), *Piezo2*flox (JAX stock #027720) and *Rosa26*-CAG-loxp-stop-loxp-tdTomato (JAX stock #007914) were obtained from Jackson Laboratory. Data reported herein have been compiled from the examination of multiple embryonic and postnatal time points for wildtype (WT), heterozygous and conditional knockout animals. Both male and female mice were used in this study; experimental comparisons that underwent naphthalene injury controlled for sex. Pregnant dams and adults were killed for experiments through CO2 asphyxiation and cervical dislocation followed by isolation of vital postnatal organs and embryos. Fetal and neonate mice (< 10 days) were put on ice until motionless and euthanized via decapitation. Timed pregnancies were confirmed through visualization of vaginal plugs with noon on the day plugs were detected designated as E0.5. Mutant and control animals were studied at E15.5, E18.5, P0, P15 and 6 weeks of age. Mice were bred and housed in the University of Wisconsin-Madison Biomedical Research Model Services Laboratory. Animal procedures were approved by the University of Wisconsin-Madison Institutional Animal Care and Use Committee and conducted in accordance with the National Institutes of Health Guide for the Care and Use of Laboratory Animals [55].

### Generation of transgenic and mutant mouse models

All offspring were genotyped using primer pairs shown in Table S1. *Shh*^cre/+^ mice were maintained by crossing male heterozygotes (*Shh*^cre/+^) with WT female littermates on a C57BL/6 J background. Mice maintained on a B6 background were used as WT controls for immunofluorescence (IF) experiments to label endogenous PIEZO protein expression patterns. *Shh*^cre/+^ mice exhibited no abnormal phenotypes and were identical to WT colony controls. *Shh*^cre/+^ males were then crossed to *ROSA26*^*CAG−LSL−tdTom/tdTom*^ females to generate *Shh*^*cre/*+^*; ROSA26*^*CAG−LSL−tdTom/*+^ (here referred to as *ROSA*^*LSL−tdTom/*+^*)* mutants. *ROSA*^*tdTom/*+^ controls were used to establish the baseline *tdTomato* levels for all experiments. *Shh*^*cre/*+^; *Piezo1*^*loxP/*+^, *Piezo2*^*loxP/*+^ males were crossed to *Piezo1*^*loxP/loxP*^, *Piezo2*^*loxP/loxP*^ females to generate conditional *Shh*^*cre/*+^; *Piezo1*^*loxP/loxP*^, *Piezo2*^*loxP/loxP*^ compound (here referred to as *Shh*^*cre/*+^; *Piezo1/2*^*cko*^) and single mutants (here referred to as *Shh*^*cre/*+^; *Piezo1*^*cko*^, *Piezo2*^*loxP/*+^ or *Shh*^*cre/*+^; *Piezo1*^loxP/+^, *Piezo2*^*cko*^). *Shh*^*cre/*+^; *Piezo1*^*loxP/*+^, *Piezo2*^*loxP/*+^ (here referred to as *Shh*^*cre/*+^*; Piezo1/2*^*loxP/*+^) and mice without the Cre allele insertion were used as littermate genotype controls. Mice lacking Cre recombination with floxed *Piezo1/2* insertions exhibited similar epithelium morphology to WT colony controls.

### Histology and immunofluorescence

Larynges and control tissues were collected at the indicated ages or timepoints following confirmation of vaginal plug and/or naphthalene administration. For embryos and P0 pups, mouse neck regions were dissected, with isolation of the entire larynx for samples > P0. All samples were immediately fixed in 4% paraformaldehyde in phosphate-buffered saline at 4 °C overnight. Samples were subsequently dehydrated in a gradient series of ethanol, treated with xylene and embedded in paraffin wax. Paraffin wax blocks were cut into serial Sects. (5 µm), dewaxed and rehydrated, heated to boiling in 10 mM citrate buffer (pH 6 or pH 9) for antigen retrieval and treated with 0.5% Triton X-100 in PBS. Sections were then stained using a standard IF protocol [56]. IF staining for PIEZO2 proteins were performed using a direct Tyramide SuperBoost™ Signal Amplification (TSA) kit (B40943, Invitrogen) followed by TrueBlack® Lipofuscin Autofluorescence Quencher (Biotium) staining following product’s published protocols. Cerebral cortical/cerebellar brain and dorsal root ganglion (DRG) tissues were isolated for positive controls for PIEZO1 and PIEZO2 antiserum, respectively (Fig. S1). Negative controls for PIEZO antibodies included antiserum + KO, antiserum + block peptide, in addition to, no applied primary antiserum. All primary and secondary antibodies used are listed in Table S2. Primary antibodies were applied overnight at 4 °C. Peptide block serum was mixed with PIEZO antibodies at 10-to-1 concentration ratio, preincubated for 60 min with rotation and then solution was applied overnight at 4 °C. Sections were incubated with DAPI (1:3000 ratio) for 10 min at RT. Slides were mounted and coverslipped with Prolong Diamond mounting media (Fisher P36970), cured flat at room temperature in the dark for 24 h, and stored at 4 °C. Each experiment was replicated at least twice for all timepoints and targets assessed.

### RNA isolation, cDNA generation and qRT-PCR

Dissected larynges from control and experimental mutants at P0 timepoint were used for RNA isolation using ReliaPrepTM RNA Tissue Miniprep System (Promega, Madison, WI, USA) following manufacturer’s protocols. For *Piezo1* and *Piezo2* mRNA transcript analysis at P0 timepoint, each control and experimental group consisted of 5 pooled larynges to maximize total RNA yield for downstream qPCR experiments. For all mRNA transcript analyses at 6wk timepoint, each control and experimental group consisted of 3 pooled larynges. One thousand ng of RNA was reverse transcribed to cDNA using reverse transcription reagents (Go Script, Promega) per manufacturer’s protocol. Expression levels of selected genes were analyzed with qPCR (Table S1). Total volume of 0.4 μl of cDNA was used per 20 μl real time qPCR reaction using Power Up Sybr Green Master Mix (Applied Biosystem, Foster City, CA, USA) and run for 40 cycles in triplicates on a 7500 Fast Real Time PCR System machine (Applied Biosystem), according to manufacturer’s instructions. Relative gene expression between control and experimental mutant larynges were normalized to β-actin (ΔCt) and control tissue (ΔΔCt).

### Naphthalene treatment

Adult 6wk mice received 0.275 mg/per gram body weight of Naphthalene (Millipore Sigma, cat# PHR1275-1G) administered with an insulin syringe 29-gauge needle dissolved in corn oil (Sigma, cat# C8267) interperitoneally. Corn oil alone injected littermates were used as control comparisons for each experimental condition with analysis at 1-, 3-, 7- and 14-dpi.

### Image acquisition

Images were acquired on a Nikon Eclipse Ti2 inverted microscope (Nikon Instruments, Inc., Belmont, CA, USA) with Nikon DS-Ri2 camera and NIS Elements software (version 5.21.01). Images were uniformly adjusted for brightness and exposure using Nikon or ImageJ software. IF staining presence, relative intensity, and subcellular and tissue-level localization were described qualitatively. Sections from experimental groups were imaged using the same exposure settings or laser power for any given antibody combination. All schematic images were created in Procreate (version 5.2.2) or BioRender.com.

### Cell count quantification

Epithelial cell counts were quantified with 40X magnification from 10 images across the length of VF ≥ 20 um apart. Images of either EdU^+^/H2AX^+^, P63^+^/SCGB1A1^+^ or P63^+^YAP^+^ stained tissues were opened in ImageJ (National Institutes of Health). Total epithelial cells, as well as EdU^+^ and P63^+^ labeled cells were enumerated using an automated counting macro “RGB fluorescent cell count v1.32.ijm” developed by Dr. David Ornelles (Wake Forest University, Winston-Salem, NC) as previously described [57, 58]. Prior to using the macro for these experiments, it was tested in comparison to manual cell counts of 40X images of VF sections from 4 serial EdU^+^ and P63^+^ stained slides. H2AX^+^ and SCGB1A1^+^ cells as well as YAP^+^ nuclei were manually counted using the built-in cell counter in ImageJ. Cytologically visible H2AX foci indicate DNA double-stranded breaks [59], with nuclei containing greater than 50% or more total area of H2AX foci considered positive.

### Statistical analysis

All data were tested for normality and equal variance using the Shapiro–Wilks and F test, respectively. A Wilcoxon rank sum test was performed to calculate significance in mRNA abundance transcript levels. To analyze WT tissue, total epithelial cell counts (DAPI) between corn oil control and naphthalene-injured VF epithelium were analyzed for comparison at each timepoint of interest (1-, 3-, 7-, 14-dpi). Proliferation and apoptosis (percentages of EdU^+^ cells and H2AX^+^ cells) as well as cell-marker indices (percentages of SCGBA1A^+^ cells, P63^+^ and YAP^+^ nuclei) were normalized to total DAPI^+^ cells for comparisons at 3 dpi timepoint. To analyze *Piezo1* allele dose effect in heterozygote versus complete knockout, P63^+^ and YAP^+^ nuclei were compared using the Wilcoxon rank sum test. One-way analysis of variance (ANOVA) was used to compare group means for relative mRNA abundance of P63^+^, YAP1^+^ and K13^+^ and K17^+^ transcripts with Tukey’s HSD post hoc test to determine pairwise comparisons. Kruskal–Wallis test was used to compare group means for relative mRNA abundance of YAP2^+^. All statistical analyses were calculated with RStudio (v. 2021.09.0 Build 351) running R 4.1.2 (R Core Team). Results are reported as either mean ± SE or median (Q2) with lower (Q1) and upper (Q3) quartile ranges. The alpha level for significance was *p* ≤ 0.05.

## Results

### PIEZO proteins exhibit selective expression in laryngeal epithelia

**Fig. 1:**
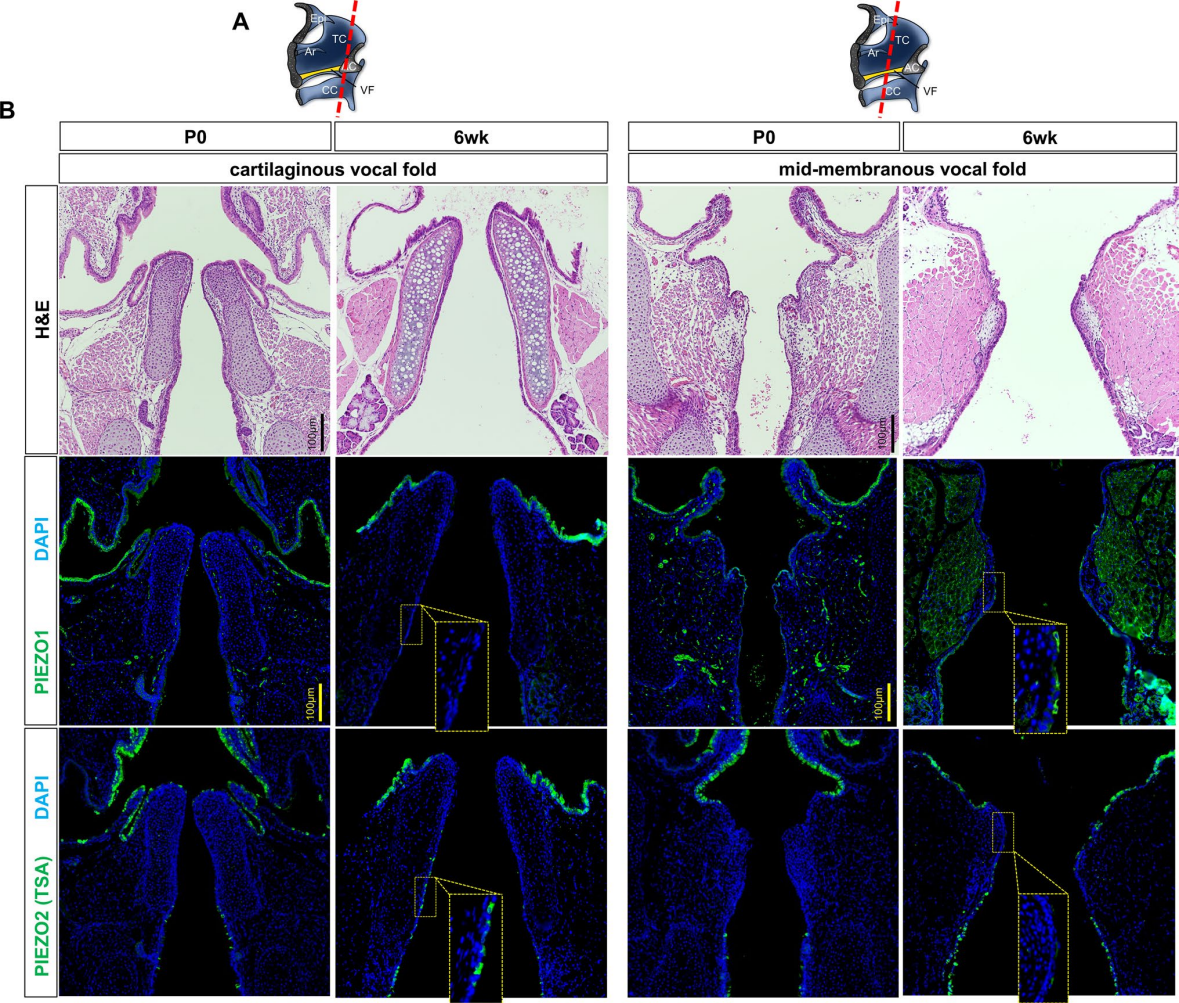
PIEZO1 & PIEZO2 exhibit selective expression to P0 & 6wk vocal fold. **a** Schematic illustrations of the sagittal view of larynx to display coronal sections (red dashed line) attained for analysis of cartilaginous and mid-membranous vocal fold epithelium. **b** H&E-stained cartilaginous and mid-membranous coronal section of vocal fold. Serial sections of PIEZO1 (green) and PIEZO2 (green) immunofluorescence protein localization in wildtype murine Bl6 vocal fold. At P0, cartilaginous vocal fold exhibit PIEZO1 selective expression to stratified, squamous epithelia of the hypopharynx, with PIEZO2 selective expression to respiratory, non-squamous epithelial cells of the hypopharynx, superior surface of arytenoids and subglottic regions. At 6wk, cartilaginous vocal fold exhibit PIEZO1 and PIEZO2 selective expression to the superior surface of arytenoid with PIEZO2 expression extending into subglottic regions. At P0, PIEZO1 expression was exhibited to epithelial cells of the mid-membranous vocal fold, however, PIEZO2 expression was noted to supra- and subglottic regions with no expression to mid-membranous vocal fold. At 6wk, PIEZO1 selective expression to differentiated, apical squamous epithelia, however, with no PIEZO2 expression. DAPI is in blue. All images 20X magnification. Scale bar represents 100 µm. *H&E* hematoxylin and eosin, *WT* wildtype, *VF* vocal fold, *P0* postnatal day 0, *TSA* tyramide signal amplification

**Fig. 2:**
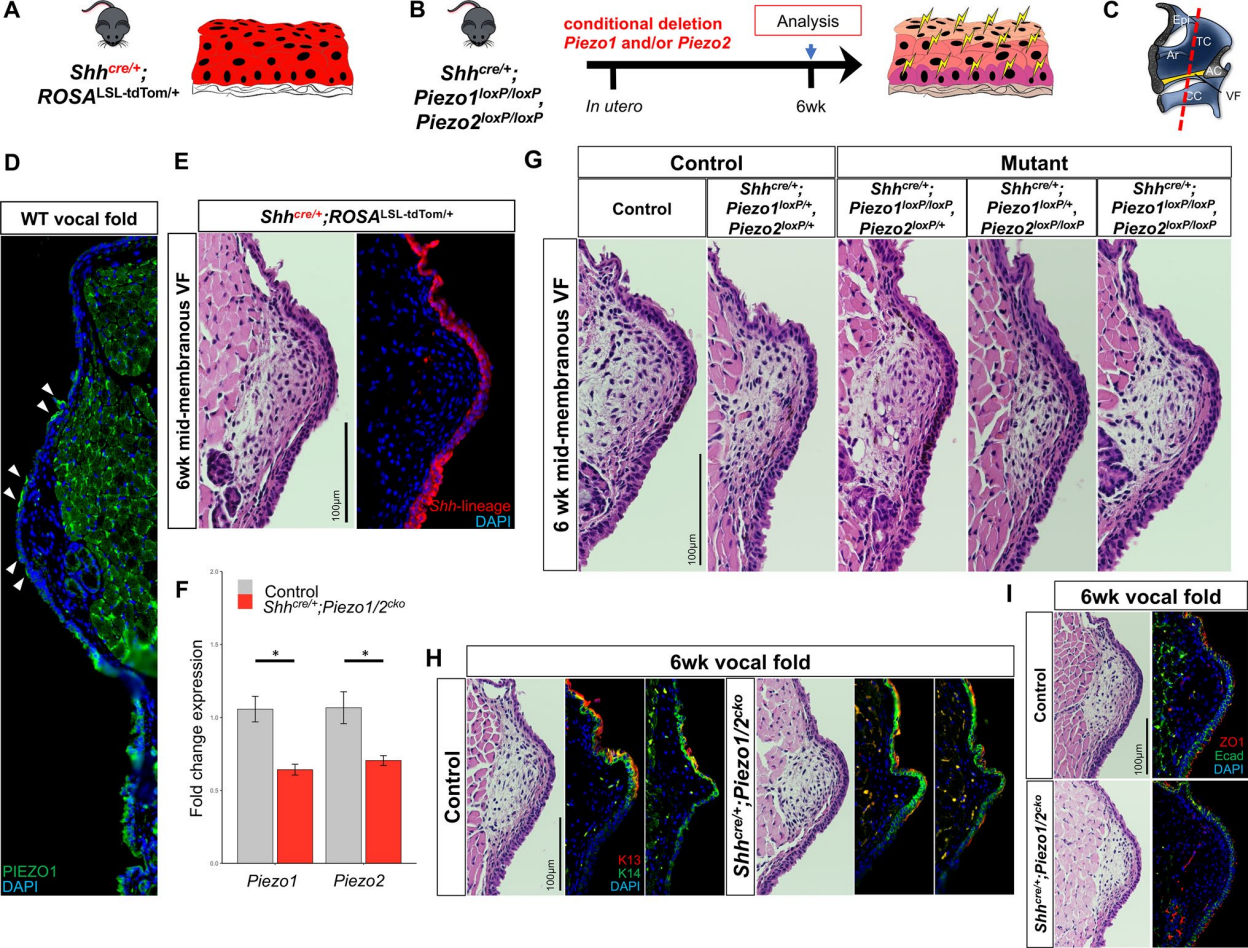
Investigation of *Shh*^*cre/*+^*;Piezo1,Piezo2* single and compound VF mutant epithelium. **a**–**c** Schematic illustrations of the experimental models and strategy for VF epithelial target-specificity and cell-specific *Piezo1Piezo2* conditional deletion denoted by yellow lightning bolts. Sagittal view of larynx to display coronal section (red dashed line) attained for analysis of mid-membranous VF epithelium shown in all images. **d** PIEZO1 protein expression localized to apical differentiated epithelial cells of the VF. **e** IF analysis using *Shh*^*cre/*+^*;ROSA*^*LSL−tdTom/*+^ mice exhibit VF epithelium-specific recombination at 6 wk timepoint. **f** Changes in mRNA transcript levels for *Piezo1* and *Piezo2* in compound mutant compared to control larynx at P0. For qPCR analysis, WT control larynges collected at P0 were used as controls for ΔΔCT data normalization. Data are presented as mean ± SEM. *n* = 5 for each genotype condition. Test: Mann–Whitney–Wilcoxon was performed to calculate significance in gene expression levels between control and compound mutant pups. **g** H&E-stained coronal sections of *Shh*^*cre/*+^*;Piezo1,Piezo2* single and/or compound mutant mice exhibit grossly normal VF epithelium compared to heterozygote and WT control at 6wk timepoint. **h** H&E-stained coronal section of mid-membranous VF. IF analysis for coronal serial sections of mid-membranous VF exhibiting normal K13 (red) and K14 (green) expression comparing control to *Shh*^*cre/*+^*;Piezo1/2cko* compound mutant. **i** IF analysis for coronal section of mid-membranous VF exhibiting normal barrier protein Ecad (green), albeit, abnormal ZO1 (red) expression comparing control to *Shh*^*cre/*+^*;Piezo1/2cko* compound mutant. DAPI is in blue. All images taken at 60X magnification. Scale bar represents 100 µm in **e**, **g**, **h**, **i**. *VF* vocal fold, *Epi* epiglottis, *TC* thyroid cartilage, *AC* arytenoid cartilage, *CC* cricoid cartilage, *Ar* aryepiglottic fold.*p value < 0.05

To investigate how ablation of *Piezo1* and *Piezo2* disrupts VF epithelia, we first assessed PIEZO1 and PIEZO2 endogenous protein expression in the larynx and VF on the background of WT control mice (Fig. 1). Two regions selected for investigation were the mid-membranous and cartilaginous arytenoid VF, known for roles in tissue oscillation [60–62] and airway protection [43, 63], respectively (Figs. 1a; 2c). Within the larynx of P0 WT mice, we detected PIEZO1 to nonkeratinized SSE of the mid-membranous and ventral VF (Fig. 1b), and keratinized SSE of the hypopharynx and esophagus (Fig. 1b; Fig. S1). PIEZO2 expression was selective to ciliated, respiratory epithelia of the hypopharynx, aryepiglottic fold, superior surface of arytenoid, inferior laryngeal epiglottis and trachea at PO, however, additionally extended into the subglottic region by 6wks (Fig. 1b). PIEZO1 expression at the 6wk timepoint was localized to apical, differentiated squamous epithelia of the VF (Figs. 1b; 2d). We next determined de novo epithelial expression of PIEZO1 and PIEZO2 proteins by staining embryonic tissue at timepoints preceding and subsequent to VF recanalization. We established PIEZO1/2 de novo epithelial cell expression patterns shortly after VF recanalization (E16.5-E17.5), denoted by no expression at E15.5, and with peak expression at E18.5 and P0 (Figs. S2&S3). We further demonstrated PIEZO2 expression abuts PGP9.5 labeled neurons in the posterior glottis and near nerve endings arborizing in the supra- and subglottis at P0 (Fig. S4).

### *Shh*^*cre/*+^; *Piezo1/2*^*cko*^ mutants develop normal VF epithelial architecture by 6wks

To determine the effect of *Piezo* removal specifically from *shh*-positive airway epithelial cells, we generated triple transgenic mice carrying the *sonic hedgehog (shh)* Cre, floxed *Piezo1* and floxed *Piezo2* alleles, referred to as *Shh*^*cre/*+^; *Piezo1/2*^*cko*^ throughout the text (Fig. 2a, b). We first verified that Cre recombinase was expressed exclusively in VF epithelial cells using a *tdTomato* reporter *(Shh*^*cre/*+^; *ROSA*^*LSL−tdTom/*+^*)* which expresses a red fluorescent protein (RFP) following Cre recombinase (Fig. 2a and e). *ROSA*^*LSL−tdTom/*+^ and no primary negative controls were used to establish baseline *tdTomato* levels and effective antiserum against RFP, respectively (Fig. S5). We then harvested P0 whole larynges from control and *Shh*^*cre/*+^*;Piezo1/2*^*cko*^ to verify effective *Piezo1/2* epithelial cell ablation, which revealed significantly reduced mRNA for both *Piezo1* (*W* = 186, *p* = 0.0001) and *Piezo2* (*W* = 174, *p* = 0.0019) (Fig. 2f). Immunofluorescence analysis validated reduced protein expression following *Piezo1* genetic ablation (Fig. S6a). Mutant combinations at P0, P15 and 6wk timepoints were collected to establish morphology following *Piezo1* and/or *Piezo2* loss. To our surprise, no epithelial morphological differences were appreciated to the mid-membranous VF across all mutant combinations at P0, P15 (Fig. S7) and/or 6wk stages (Fig. 2g). IF analysis confirmed normal cytokeratin differentiation expression of SSE markers K13 and K14 in *Shh*^*cre/*+^; *Piezo1/2*^*cko*^ compound mutant compared to control tissue (Fig. 2h), albeit, downregulated ZO1 to apical epithelium following *Piezo* ablation (Fig. 2i). To further investigate epithelial structural changes, we stained for MUC1, an important mucin previously characterized in human VF epithelium [64]. Interestingly, we found overexpression of MUC1 following *Piezo1* deletion, which suggests the VF epithelium compensates via downregulating ZO1 and upregulating MUC1 production ultimately priming signaling cascades for repair strategies (Fig. S8).

### NAPH-induced injury results in hyperplasia to WT VF epithelium

**Fig. 3:**
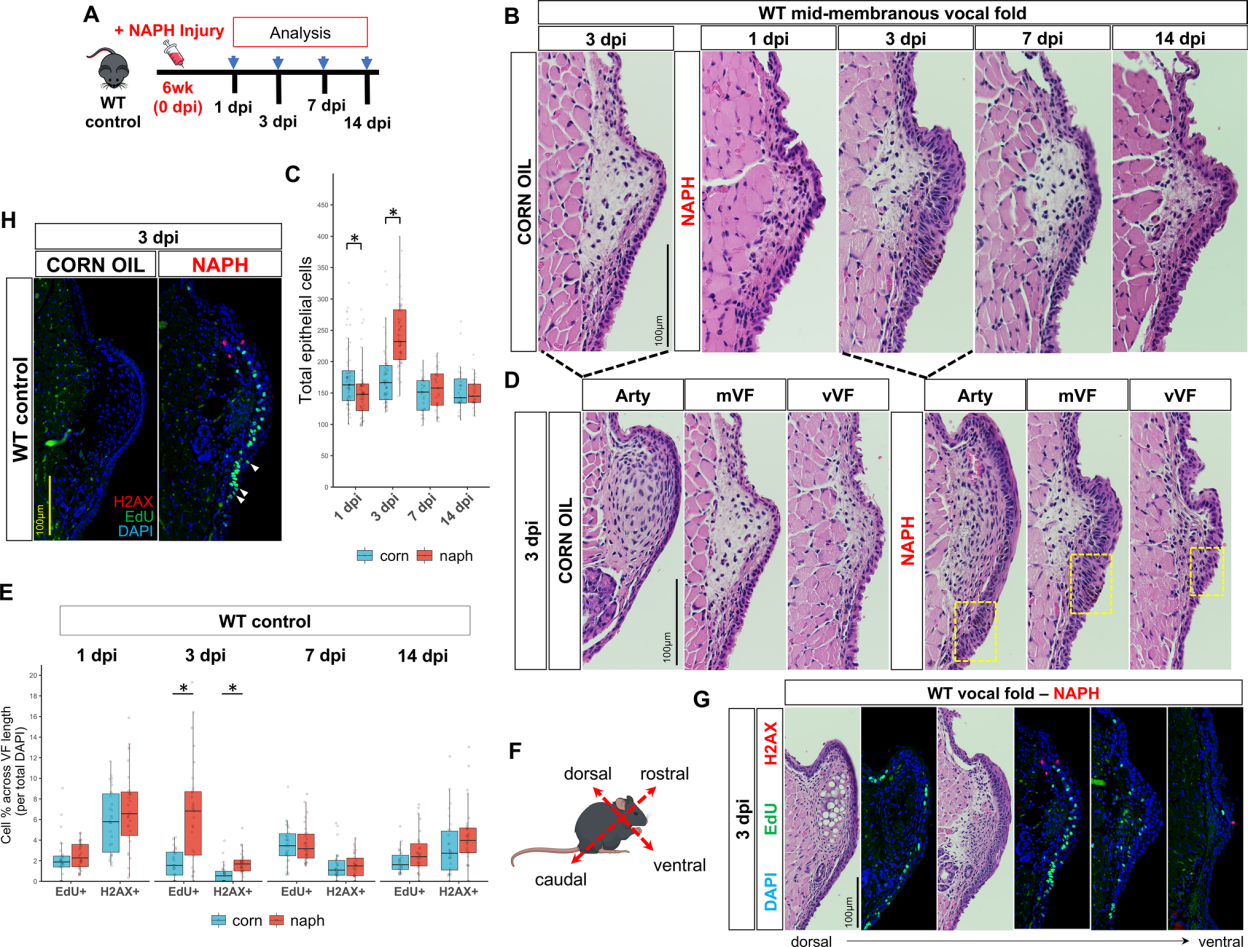
NAPH-injured WT VF epithelium exhibits epithelial hyperplasia with increased cell cycle activation of EdU + /H2AX + by 3 dpi. **a** Schematic illustration of the analysis for VF epithelial NAPH injury. **b** H&E-stained VF sections from 3dpi control (left) compared to 1, 3, 7, and 14 dpi following NAPH treatment. **c** Quantification of total epithelial cells comparing corn oil controls versus naphthalene-injured VF sections at 1, 3, 7, and 14 dpi exhibit significantly increased total epithelia by 3dpi for NAPH-injured group. *n* = 6 for each treatment condition per timepoint. **d** H&E-stained serial sections of WT VF across the dorsal–ventral plane at 3dpi for corn oil control versus NAPH-injured. Yellow boxes indicate subglottic regions with abnormal epithelial morphology characterized by irregular apical and basolateral polarity. **e** Quantification of EdU + DNA synthesis (cell proliferation) and H2AX + DNA damage (double-stranded breaks) in WT controls for corn oil versus NAPH-injured VF sections at 1, 3, 7, 14dpi exhibit significantly increased EdU + H2AX + at 3dpi only. n = 3 for each treatment condition and timepoint per target. **f** Schematic illustration of murine anatomic axis. **g** IF analysis of VF serial sections along the dorsal–ventral (left-to-right) plane for EdU + (green) and H2AX + (red) exhibit increased EdU + marked epithelial cell proliferation to the dorsal VF. **h** Highly concentrated EdU + proliferative cells are exhibited to unique subglottic cell compartment in NAPH-injured group only with white arrows denoting suprabasal EdU + cells undergoing S-phase DNA synthesis. DAPI is in blue. Images taken at 60X in **b**, **d** and 40× magnification in **g**, **h**. Scale bar represents 100 µm in **b**, **d**, **g**, **h**. Data are presented as the mean (Q2) with lower (Q1) and upper (Q3) quartile ranges. Test: Student’s *t* test or Mann–Whitney–Wilcoxon was performed for all target analyses at each timepoint of interest in **c**, **e**. *WT* wildtype, *NAPH* naphthalene, *IF* immunofluorescence, *dpi* days post-injury, *Arty* arytenoid, *mVF* mid-membranous vocal fold, *vVF* ventral vocal fold, *LP* lamina propria. *p < 0.05

NAPH exposure in mice results in airway epithelium-specific injury, which has proven valuable for study of regeneration in distal lung and tracheal airways [48–51, 65, 66]. Adult 6wk control, *Shh*^*cre/*+^*;Piezo1/2*^*loxP/*+^ haploinsufficient and *Shh*^*cre/*+^*;Piezo1*^*cko*^*,Piezo2*^*loxP/*+^ knockout animals were treated with NAPH or vehicle-treated (corn oil) control and sacrificed at 1-, 3-, 7-, and 14-dpi (Fig. 3a). To test feasibility of NAPH chemical exposure to result in VF epithelial injury, WT controls were used in our initial analyses. No epithelial injury was observed in WT controls on day 1 following NAPH exposure, however, by 3 dpi, WT VF epithelium exhibited hyperplasia with increased stratification of squamous cells and with significantly increased total epithelial cells compared to corn oil uninjured controls (173 ± 45 corn vs. 242 ± 52 naph, *p* < 0.0001) (Fig. 3b, c). This increased stratification and resulting hyperplasia was noted across the full length of the VF (Fig. 3d). IF analysis corroborated histological findings, demonstrating increased EdU^+^ epithelia (1.71 ± 1.35 corn vs. 6.56 ± 5.01 naph, *p* < 0.0001) and H2AX^+^ epithelia (0.67 ± 0.92 corn vs. 1.74 ± 1.08 naph, *p* < 0.0001), compared to controls at 3 dpi only (Fig. 3e). Hyperplastic changes were largely resolved by 7 dpi (Fig. 3b), with no differences in epithelial cell counts (148 ± 26 corn vs. 154 ± 31 naph, *p* = 0.1988) (Fig. 3c). Abnormal cell morphology was localized to the VF subglottic region (Fig. 3d), which was defined as below the point where squamous epithelium of the VF reaches its inferior extent (a.k.a. squamociliary junction). Further characterization of serial sections revealed increased EdU^+^ epithelia along the dorsal–ventral plane to the arytenoid and mid-membranous regions compared to ventral sections (Fig. 3g), with increased concentrated labeling of EdU + cell proliferation to the subglottic region of the mid-membranous fold (Fig. 3h).

**Fig. 4:**
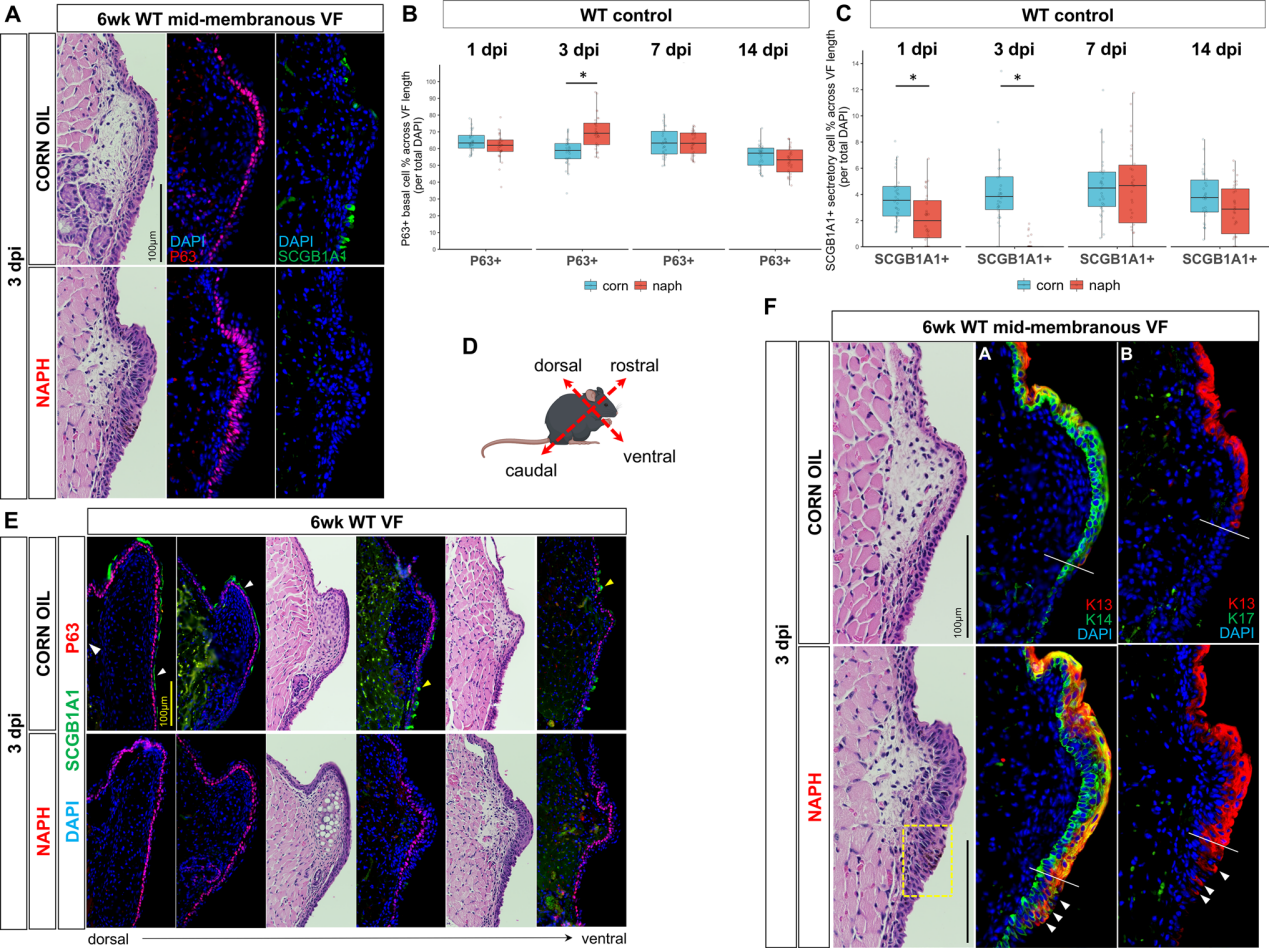
Characterization of NAPH injury model in wildtype controls at 3 dpi. **a** IF analysis of basal cell marker P63 (red) and secretory cell marker SCGB1A1 (green) in WT VF comparing normal (corn oil) versus injured (NAPH) exhibits secretory cell ablation with expansion of P63 + basal cell compartment following NAPH exposure. DAPI is in blue. **b** Quantification of P63 + marked basal cells exhibit significantly increased basal cell compartment expansion at 3dpi only. **c** Quantification of SCGB1A1 + secretory marked cells in WT controls versus NAPH-injured VF sections exhibit transient sensitivity to NAPH exposure with significant cell decrease by 1 dpi, near complete ablation by 3 dpi, albeit, population rebound by 7 dpi with no differences at 7 or 14 dpi. **d** Schematic illustration of murine anatomic axis. **e** IF analysis of basal cell marker P63 (red) and secretory cell marker SCGB1A1 (green) in corn oil control and NAPH-treated WT VF along the dorsal–ventral plane. White arrows indicate SCGB1A1 + squamous cells, while yellow arrows indicate SCGB1A1 + cuboidal cells. **f** H&E-stained coronal sections of WT VF at 6 wk timepoint following either corn oil control or NAPH exposure results in VF epithelial hyperplasia with abnormal subglottic cell morphology (yellow box). IF analysis revealed overexpression of K13 and K14 following NAPH injury. White lines indicate squamous-to-ciliated epithelial cell junction (i.e. squamociliary), and white arrows indicate abnormal, ectopic K13 expression to non-squamous epithelia of the subglottis following NAPH injury compared to corn oil controls. Image in **a** and **f** 60× and **e** 40× magnification. Scale bar represents 100 µm in **a**, **f** and **e**. Data are presented as the mean (Q2) with lower (Q1) and upper (Q3) quartile ranges. n = 3 for each treatment condition and timepoint per target. Test: Student’s *t* test or Mann–Whitney–Wilcoxon was performed for all target analyses at each timepoint of interest. *WT* wildtype, *K* keratin, *VF* vocal fold, *IF* immunofluorescence, *dpi* days post-injury, *p < 0.05

To determine potential mechanism for NAPH-induced epithelial injury and resulting hyperplasia, VF sections were stained for P63 and SCGB1A1, indices for basal cell progenitor and secretory cells, respectively. NAPH-injured WT VF epithelium exhibited expansion to P63^+^ cells within the basal compartment (Fig. 4a). Cell count quantification revealed significantly increased P63^+^ cells following NAPH injury at 3 dpi only (58.0 ± 8.14 corn vs. 70.2 ± 9.85 naph, *p* < 0.0001) (Fig. 4b). IF analysis also exhibited ablation of SCGB1A1^+^ secretory cells to the mid-membranous fold at 3 dpi compared to corn oil controls (Fig. 4a). SCGB1A1^+^ secretory cells were demonstrated across the full length of VF in WT corn oil controls in both squamous and cuboidal epithelia (Fig. 4e). Furthermore, quantification comparing injured to uninjured controls at each timepoint revealed transient epithelial sensitivity to NAPH with significant cell decrease by 1 dpi (3.70 ± 1.79 corn vs. 2.21 ± 1.89 naph, *p* = 0.003), significant near complete ablation by 3 dpi (4.33 ± 2.61 corn vs. 0.21 ± 0.49 naph, *p* < 0.0001), albeit, population rebound by 7 dpi (4.67 ± 2.45 corn vs. 4.54 ± 3.19 naph, *p* = 0.8608) and no significance differences at either 7 or 14 dpi (3.78 ± 1.9 corn vs. 2.83 ± 1.93 naph, *p* = 0.0643) (Fig. 4c). Lastly, we stained markers for K13 and K14, known to be important for maturation and differentiation of murine SSE [26, 56, 67], along with the stress cytokeratin marker K17. Most interestingly, expression of K13 extended into the subglottic region with apparent positivity to non-squamous epithelia at the squamociliary junction (Fig. 4f), with K14 overexpression extending into the tracheal lower airway following NAPH injury compared to controls (Fig. S9). No K17 expression was noted in either treatment group (Fig. 4f).

### *Piezo1* ablation results in increasingly severe VF epithelial defects

**Fig. 5:**
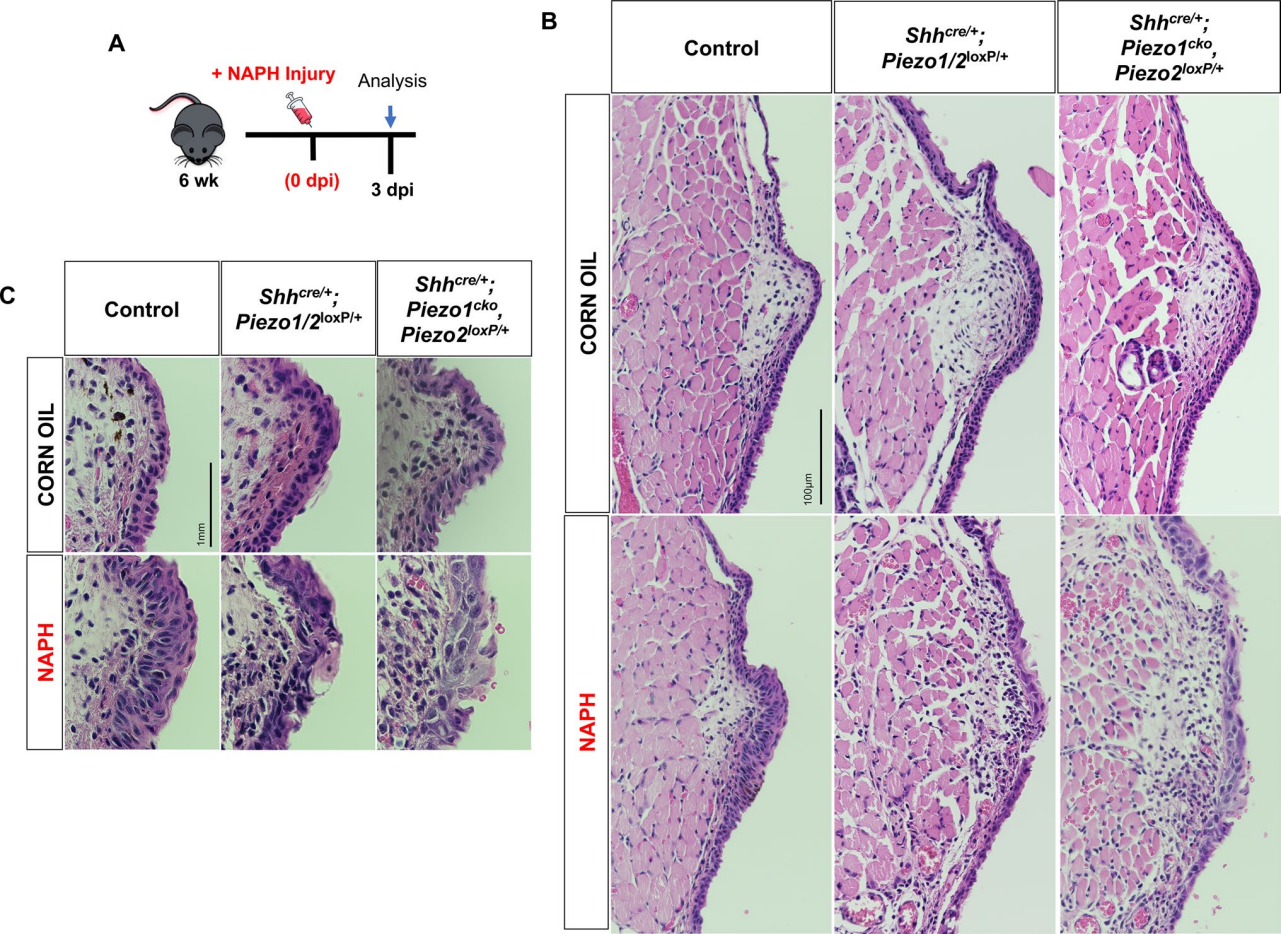
*Piezo1* ablation results in increasingly severe VF epithelial defects following NAPH injury at 3 dpi. **a** Schematic illustration of the analysis for VF epithelial NAPH injury. **b** NAPH injury induces epithelial defects at 3dpi in both *Shh*^*cre/*+^*;Piezo1/2*^*loxP/*+^ and *Shh*^*cre/*+^;*Piezo1*^*cko*^*,Piezo2*^*loxP/*+^ mutants characterized by loss of stratification, disorganized/large squamous cells with polymorphous nuclei, irregular nuclear borders and large amounts of cytoplasm with indistinct cell borders. **c** 150× highly magnified images comparing corn oil to NAPH-injured VF epithelium medial edge. Images taken at 40× in **(b)** and 150× magnification in **c**. Scale bar represents 100 µm in **b** and 1 mm in **c**. *dpi* days post-injury, *WT* wildtype, *NAPH* naphthalene

**Fig. 6:**
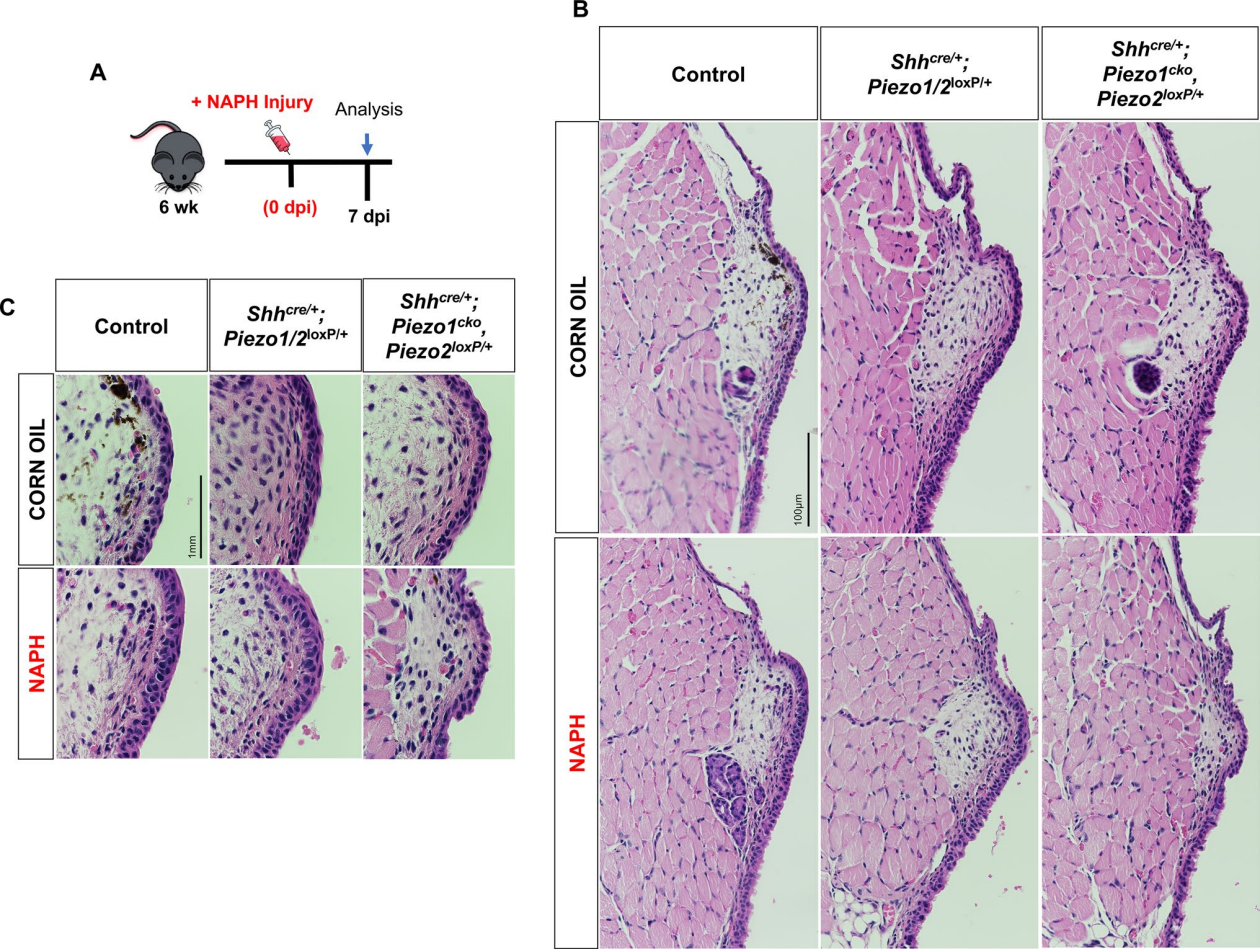
WT and *Piezo1* mutant epithelium exhibits restoration of cell defects following NAPH exposure at 7 dpi. **a** Schematic illustration of the analysis for VF epithelial NAPH injury. **b** By 7 dpi, aberrant epithelial morphology is restored with architecture similar to corn oil controls for all genotype conditions. **c** 150× highly magnified images comparing corn oil to NAPH-injured VF epithelium medial edge. Images taken at 40× in **b** and 150× magnification in **c**. Scale bar represents 100 µm in **b** and 1 mm in **c**. *dpi* days post-injury, *WT* wildtype, *NAPH* naphthalene

We hypothesized that following NAPH insult and loss of *Piezo1* to apical VF epithelial cells that we would observe epithelial remodeling defects. Given data from WT controls, we expected severe injury to occur by 3 dpi. Consistent with our hypothesis, we noted a *Piezo1* allele dose effect such that following *Piezo1* loss mutant epithelium exhibited increasingly severe cell defects compared to NAPH-treated control and/or *Shh*^*cre/*+^*;Piezo1/2*^*loxP/*+^ heterozygotes (Fig. 5a–c). Phenotypic abnormalities to *Shh*^*cre/*+^*;Piezo1*^*cko*^*,Piezo2*^*loxP/*+^ mutants were characterized by loss of stratification, disorganized/large squamous cells with polymorphous nuclei, irregular nuclear borders and large amounts of cytoplasm with indistinct cell borders (Fig. 5b, c). Epithelium defects across all genotype conditions were resolved by 7 dpi with no differences to corn oil controls, characterized by re-epithelization and complete restoration (Fig. 6a–c).

### *Piezo1* modulates self-renewal via repressive effects on *p63* and YAP subcellular localization

**Fig. 7:**
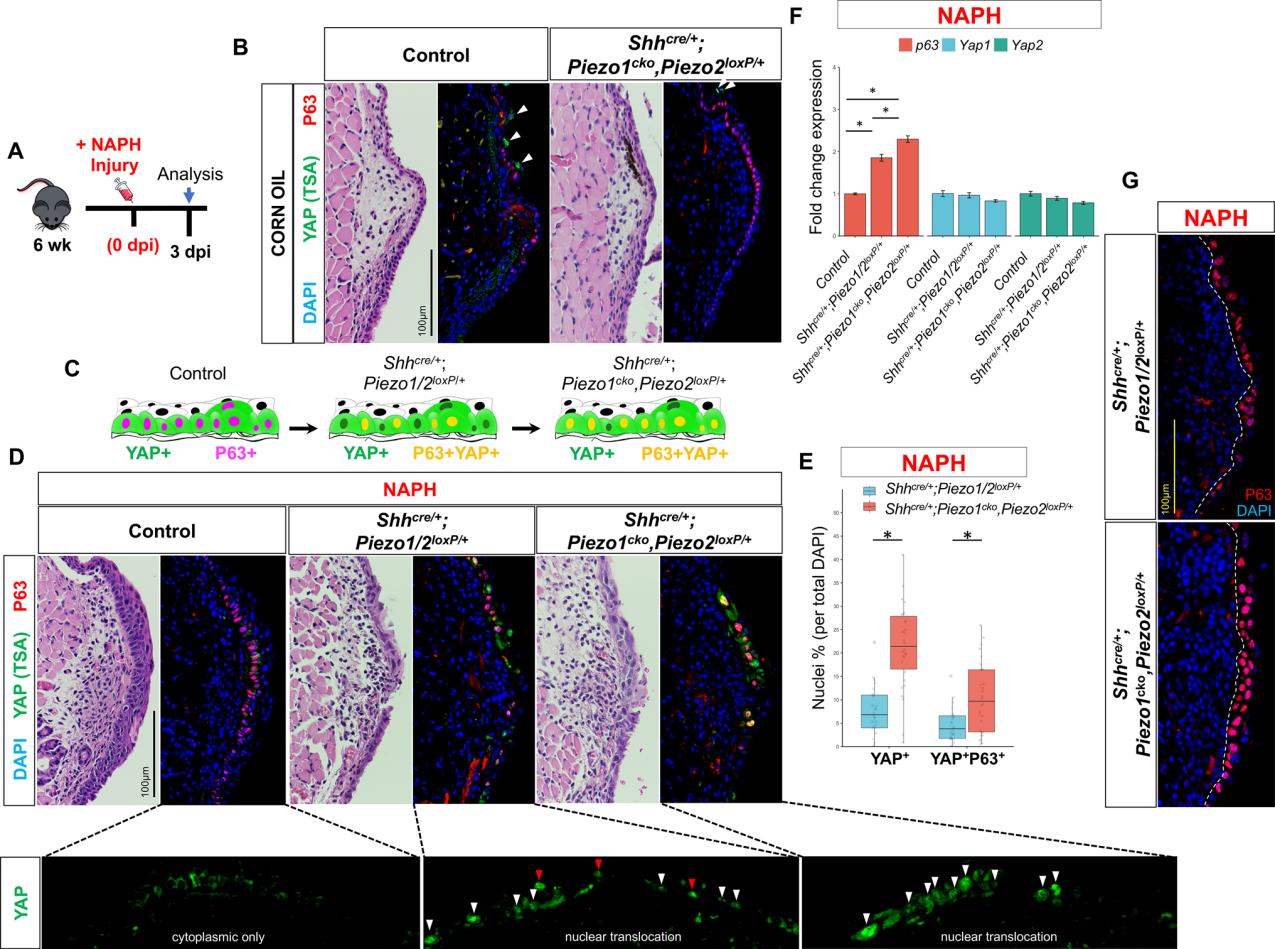
*Piezo1* loss results in nuclear YAP with co-localization of YAP^+^P63^+^ to VF basal epithelia. **a** Schematic illustration of the analysis for VF epithelial NAPH injury. **b** IF analysis exhibiting corn oil control and *Shh*^*cre/*+^*;Piezo1*^*cko*^*,Piezo2 *^*loxP/*+^ mutant VF epithelium expresses mainly cytoplasmic YAP with very few cells exhibiting nuclear expression mainly localized to apical cells denoted by white arrows. **c** Hypothetical schema showing that, following *Piezo1* loss, basal cells increase co-localization of YAP and P63 markers. **d** IF analysis of total YAP (green) and basal cell marker P63 (red) exhibit cytoplasmic-to-nuclear YAP translocation expression pattern comparing controls to *Shh*^*cre/*+^*;Piezo1/2*^*loxP/*+^ and *Shh*^*cre/*+^*;Piezo1*^*cko*^*,Piezo2 *^*loxP/*+^ mutants. Red arrows represent YAP + P63-, while white arrows represent YAP + P63 + nuclear co-localization. DAPI is in blue. **e** Quantification reveals significantly different percentage of nuclear YAP + and YAP + P63 + marked cells comparing *Shh*^*cre/*+^*;Piezo1/2*^*loxP/*+^ to *Shh*^*cre/*+^*;Piezo1*^*cko*^*,Piezo2 *^*loxP/*+^ mutants following *Piezo1* loss. Data are presented as the mean (Q2) with lower (Q1) and upper (Q3) quartile ranges. *n* = 3 for each genotype condition. **f** Quantitative-PCR analysis shows significantly increased *p63* mRNA expression following *Piezo1* haploinsufficient and null allele loss. No significant change in mRNA expression of *Yap1* and/or *Yap2* target genes following *Piezo1* loss. Data are presented as mean ± SEM. *n* = 3 digested larynges for each genotype condition. **g** IF analysis of basal cell marker P63 (red) exhibit *Piezo1* allele dose effect via increased expression with nuclear cell hypertrophy following *Piezo1* loss. White dashed line indicates epithelium and LP transition boundary. All images taken at 60× magnification. Scale bar represents 100 µm. Test: One-Way ANOVA with Tukey’s HSD post-hoc for *p63* and *Yap1* analysis; Kruskal–Wallis for *Yap2* analysis in **f** and Mann–Whitney-Wilcoxon in **e**. *VF* vocal fold, *IF* immunofluorescence, *dpi* days post-injury, *NAPH* naphthalene. *p < 0.05

Previous findings from our lab have demonstrated the importance of Hippo/YAP pathway for VF epithelium morphogenesis [68]. Moreover, *Piezo1* and *Piezo2* have been implicated as upstream components for signal transduction pathways resulting in YAP transcriptional activity for stem cell differentiation [69]. Mice were investigated at 3dpi due to the appreciated phenotypic differences (Fig. 7a). Adult VF epithelium expresses mainly cytoplasmic YAP with very few cells exhibiting nuclear expression mainly localized to apical cells (Fig. 7b) and indicating a role in epithelial cell stratification [68]. Prior work has also revealed a role for both P63 and YAP in epidermal basal SC proliferation and epithelial stratification [70–73], with additional evidence implicating their genetic interaction in the airway epithelium [74]. We reasoned that injury to the VF may result in increased YAP^+^P63^+^ epithelial cells with nuclear YAP translocation in our controls which exhibited hyperplasia and increased keratin expression. We also hypothesized analogous loss of YAP expression following *Piezo1* loss-of-function in our mutant epithelium given prior work [69, 75]. IF analysis revealed upregulated YAP in control epithelium following NAPH injury at 3 dpi, albeit, with grossly cytoplasmic expression localized to the basal cell compartment (Fig. 7c, d). More intriguing was the translocation to nuclear YAP expression in *Shh*^*cre/*+^*;Piezo1/2*^*loxP/*+^ *and Shh*^*cre/*+^*;Piezo1*^*cko*^*,Piezo2*^*loxP/*+^ injured mutant VF epithelium (Fig. 7c, d). We also appreciated increased co-localization of nuclear P63 and YAP expression in *Shh*^*cre/*+^*;Piezo1*^*cko*^*,Piezo2*^*loxP/*+^ mutant VF epithelia (Fig. 7c, d). Cell count quantification revealed significantly increased nuclear YAP^+^ (7.87 ± 5.78 *Shh*^*cre/*+^*;Piezo1/2*^*loxP/*+^ vs. 22.1 ± 10.2 *Shh*^*cre/*+^*;Piezo1*^*cko*^*,Piezo2*^*loxP/*+^, p < 0.0001) and YAP^+^P63^+^ (4.72 ± 3.97 *Shh*^*cre/*+^*;Piezo1/2*^*loxP/*+^ vs. 10.4 ± 7.46 *Shh*^*cre/*+^*;Piezo1*^*cko*^*,Piezo2*^*loxP/*+^, *p* = 0.002) VF epithelia following *Piezo1* loss in NAPH-exposed groups (Fig. 7e). Additionally, we performed quantitative-PCR analysis for *p63, Yap1,* and *Yap2* transcripts to assess mRNA abundance across genotype comparisons. We found *p63* mRNA exhibited an inverse relationship following *Piezo1* loss in NAPH-exposed groups, such that *p63* expression increased in a linear manner from control to *Shh*^*cre/*+^*;Piezo1*^*cko*^*,Piezo2*^*loxP/*+^ mutant VF epithelium (Fig. 7f). One-way ANOVA test revealed significant group differences (*F*(2,6) = 101, *p* < 0.0001), with Tukey-adjusted HSD post hoc tests exhibiting significantly different *p63* mRNA expression (*p* < 0.0001) for all genotype pairwise comparisons. This supports *Shh*^*cre/*+^*;Piezo1*^*cko*^*,Piezo2*^*loxP/*+^ histologic findings of nuclear hypertrophy, with IF analysis corroborating more robust P63 nuclear expression following *Piezo1* loss compared to heterozygote epithelium (Fig. 7g). There were no group differences in *Yap1* (*F*(2,6) = 2.607, *p* = 0.153) and/or *Yap2* (*H*(2) = 4.3556, *p* = 0.1133) mRNA abundance across genotype conditions (Fig. 7f). While overall *Yap* mRNA transcript levels are unchanged, the clear shift from cytoplasmic-to-nuclear YAP protein localization suggests posttranslational modification, which may have a functional effect on *p63* resulting in appreciated basal progenitor cell proliferation and epithelial stratification events.

### *Piezo1* loss alters differentiation programs for VF remodeling events

**Fig. 8:**
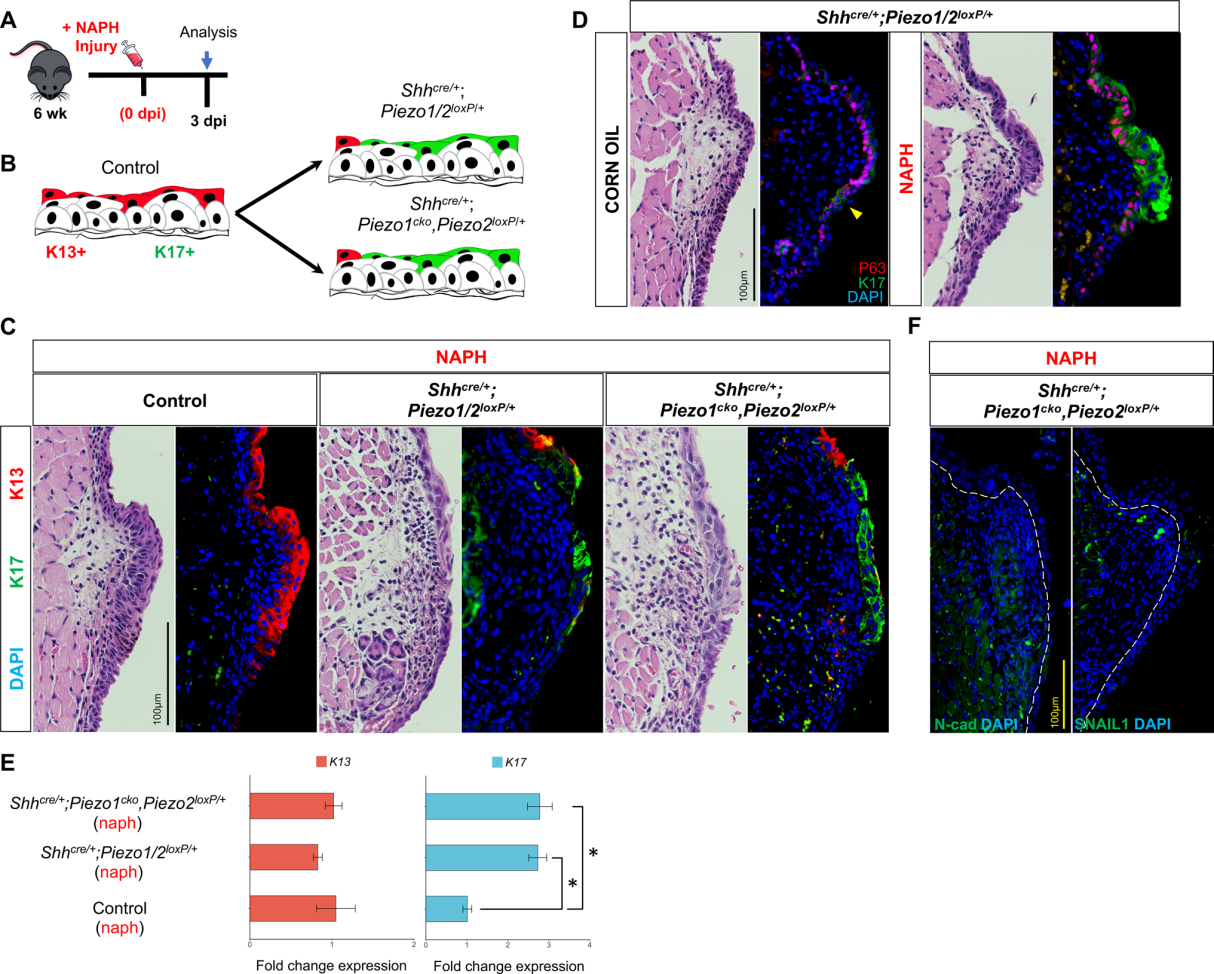
*Piezo1* loss results in ectopic K17 expression to VF epithelial cells following NAPH-injury. **a** Schematic illustration of the analysis for VF epithelial NAPH injury. **b** Hypothetical schema showing that, following *Piezo1* allele loss, VF stratified squamous epithelial cells ectopically express K17. **c** IF analysis of K17 (green) and K13 (red) exhibit ectopic switch to K17 expression pattern comparing control to *Shh*^*cre/*+^*;Piezo1/2*^*loxP/*+^ and *Shh*^*cre/*+^*;Piezo1*^*cko*^*,Piezo2*^*loxP/*+^ mutants. DAPI is in blue. **d** IF analysis of *Shh*^*cre/*+^*;Piezo1/2*^*loxP/*+^ mutant epithelium comparing corn oil controls to NAPH-injured mid-membranous section displays ectopic K17 focal emergence to squamociliary junction in absence of injury with spread to entire epithelium following injury. **e** Quantitative-PCR analysis shows significantly increased *K17* mRNA expression following *Piezo1* haploinsufficient and null allele loss. No significant change in *K13* mRNA expression following *Piezo1* loss. Data are presented as mean ± SEM. *n* = 3 digested larynges for each genotype condition. **f** IF analysis for markers of epithelial-to-mesenchymal transition N-cadherin (green) and SNAIL1 (green) exhibit lack of protein expression in *Shh*^*cre/*+^*;Piezo1*^*cko*^*,Piezo2*^*loxP/*+^ mutants following NAPH-injury. White dashed line indicates epithelium and LP transition boundary. Image in **c**, **d** 60× and **f** 40× magnification. Scale bar represents 100 µm in **c**, **d**, **f**. Test: One-Way ANOVA with Tukey’s HSD post hoc for *K13* and *K17* analysis in **d**. *dpi* days post-injury, *IF* immunofluorescence, *NAPH* naphthalene, *VF* vocal fold, *K* keratin. *p < 0.05

K17, not normally expressed in healthy VF epithelium, is known for its role in wound healing and DNA damage [76–78]. Prior work has also established reciprocal effects of K17 emergence with K13 loss in head and neck squamous cell carcinoma (SCC) [79–81]. We therefore postulated that aberrant K13/K17 expression may be partly contributing to abnormal stratification events appreciated in our *Piezo1* ablated epithelium. To test this, we stained control, *Shh*^*cre/*+^*;Piezo1/2*^*loxP/*+^ and *Shh*^*cre/*+^*;Piezo1*^*cko*^*,Piezo2*^*loxP/*+^ mutant tissue at 3 dpi following NAPH or corn oil exposure (Fig. 8a). K13/K17 expression patterns were consistent with prior characterization data such that K13 was increased after injury localized to apical VF epithelia with a lack of K17 expression to VF epithelium in either treatment condition (Fig. 4f; Fig. 8b, c). Interestingly, K17 expression was exhibited to the subglottis at the squamociliary junction in corn oil uninjured *Shh*^*cre/*+^*;Piezo1/2*^*loxP/*+^ mutant epithelium increasing to surrounding VF SSE following NAPH injury (Fig. 8d). *Shh*^*cre/*+^*;Piezo1/2*^*loxP/*+^ mutant larynx displayed preferential K17 expression to squamous epithelia of the VF, as well as squamous and intermediate epithelia of the laryngeal surface of the epiglottis (Fig. S10). Further investigation revealed what seemed to be reciprocal expression patterns in *Shh*^*cre/*+^*;Piezo1/2*^*loxP/*+^ haploinsufficient and *Shh*^*cre/*+^*;Piezo1*^*cko*^*,Piezo2*^*loxP/*+^ mutant VF epithelium following injury, whereas loss of K13^+^ epithelia resulted in increased ectopic K17^+^ epithelia (Fig. 8c). However, quantitative-PCR analysis confirmed no difference for *K13* mRNA across genotype conditions (*F*(2,6) = 0.63, *p* = 0.565), albeit, group differences were noted comparing *K17* mRNA abundance across conditions (*F*(2,6) = 20.168, *p* = 0.002). Tukey-adjusted post hoc pairwise tests revealed significantly increased *K17* mRNA comparing controls to *Shh*^*cre/*+^*;Piezo1/2*^*loxP/*+^ (*p* = 0.0039) and *Shh*^*cre/*+^*;Piezo1*^*cko*^*,Piezo2*^*loxP/*+^ (*p* = 0.0034) mutants following NAPH injury (Fig. 8e). To further explore if aberrant K13/K17 differentiation was related to beginning stages for squamous cell metaplasia, we stained for SNAIL1 and N-cadherin [82–84]. Results were negative for both markers in *Shh*^*cre/*+^*;Piezo1*^*cko*^*,Piezo2*^*loxP/*+^ mutant epithelium ruling out the possibility of epithelial-to-mesenchymal transition (EMT) (Fig. 8f).

### Barrier integrity is compromised up to 14 dpi in *Shh*^*cre/*+^*;Piezo1*^*cko*^*,Piezo2*^*loxP/*+^ mutant VF epithelium

**Fig. 9:**
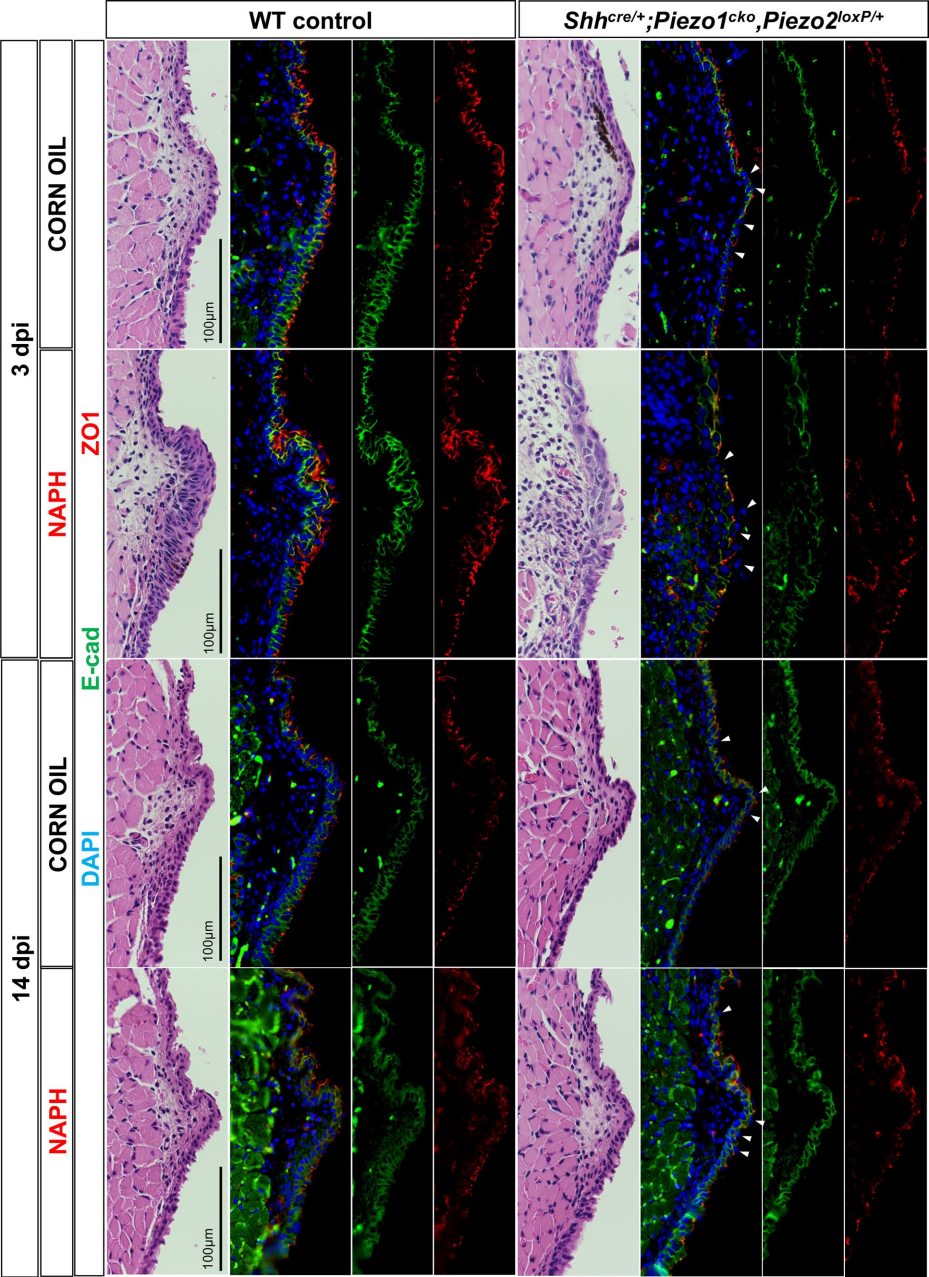
VF epithelium exhibits compromise to ZO1-marked apical epithelia following *Piezo1* loss regardless of injury status. By 3 dpi, IF analysis exhibited increased adheren and tight junctions marked by E-cadherin (green) and ZO1 (red), respectively, in WT VF epithelium following NAPH exposure with tight junctions mislocalized, extending into the basal stratum. At 3dpi, *Shh*^*cre/*+^*;Piezo1*^*cko*^*,Piezo2*^*loxP/*+^ mutant epithelium exhibited discontiguous ZO1-marked tight junctions indicated by white arrows in either corn oil control and NAPH-injured conditions, while E-cad-marked adheren junctions were disrupted only following NAPH-induced injury. By 14 dpi, mutant epithelium continued to exhibit compromised barrier integrity indicated by discontiguous ZO1 expression patterns following *Piezo1* loss. DAPI is in blue. All images taken at 60X magnification. Scale bar represents 100 µm. *dpi* days post-injury, *NAPH* naphthalene

Damage to the epithelial stratum results in barrier function compromise, associated with acute phases of VF injury and ensuring tissue remodeling [15, 17, 25, 85–89]. We performed IF analysis at 3- and 14-dpi for adheren junction marker, E-cadherin and tight-junction marker, ZO1. At 3 dpi, we found normal E-cadherin cell–cell expression with localized ZO1 expression to suprabasal epithelia in corn oil controls (Fig. 9). However, NAPH-injured WT VF exhibited increased E-cadherin and ZO1 to hyperplastic epithelium with mislocalized ZO1 penetrating to the basal cell layer (Fig. 9). As expected, NAPH-injured *Shh*^*cre/*+^*;Piezo1*^*cko*^*,Piezo2*^*loxP/*+^ mutant VF epithelium displayed discontiguous expression patterns of E-cadherin and ZO1 resulting from epithelium defects at 3 dpi. Interestingly, ZO1 discontiguous expression was also noted in corn oil treated *Shh*^*cre/*+^*;Piezo1*^*cko*^*,Piezo2*^*loxP/*+^ control epithelium (Fig. 9). By 14 dpi, corn oil control and NAPH-injured *Shh*^*cre/*+^*;Piezo1*^*cko*^*,Piezo2*^*loxP/*+^ mutant VF epithelium continued to exhibit discontiguity of ZO1 expression to apical epithelia, suggesting continued structural compromise to restored epithelium (Fig. 9).

## Discussion

We have leveraged the recent discovery of *Piezo1* [29], a mechanosensitive channel known to regulate the life cycle of epithelial cells [31–33], to test the hypothesis that *Piezo1* is a critical mechanoprotein for VF SSE and repair pathways for acute VF remodeling events. PIEZO expression displayed selectivity to distinct laryngeal epithelia, such that PIEZO1 was expressed to differentiated, apical SSE of the VF, hypopharynx and esophagus, while PIEZO2 expression was restricted to respiratory and intermediate epithelia of supra- and subglottic regions. Cell expression patterns are likely the result of evolved function for various airway epithelium. Human studies have shown that the mid-membranous VF is largely unresponsive to stimulation of the mucosa, suggesting lack of laryngeal adductor reflex (LAR) sensory receptors, and a physiological advantage due to the high biomechanical loads endured during phonation [43, 63]. On the contrary, PIEZO2-expressing epithelia predominated in LAR sensory hotspots [1, 43, 63, 90, 91], and abutted PGP9.5-labeled arborizing nerve fibers (Fig. S4), suggesting a possible role for laryngeal mechanosensation. Direct links to *Piezo2* mechanosensory function in the larynx, however, has not been established. Contrary to expectations, *Piezo1* was dispensable for proper specification and maturation of developing VF epithelium with normal histology and cytokeratin differentiation in *Shh*^*cre/*+^*;Piezo1/2*^*cko*^ compound mutant epithelium at the 6wk timepoint. We suspect that during normal development and epithelial homeostasis of the murine VF that the mechanical environment is not elevated enough to result in *Piezo1* activation, thus lacking aberrant pathway signaling following *Piezo1* loss. While previous evidence suggests that mice are capable of VF vibration [92–94], the primary mode of vocal communication is done using ultrasonic vocalization (USV), which is produced in the larynx [95–97]. Audible cries, also attributed to VF vibration, is produced in laboratory mice, albeit, to a limited extend with USV predominating for close-distance mating and social contexts [95]. Given the lack of impact collisional forces during VF vibration in these settings, we suspect the mechanical challenge to VF mucosa is quite low in laboratory-housed mice. In other words, the reduced biomechanical challenge to VF mucosa in laboratory-housed mice may explain why we see limited *Piezo1* localized expression in sparse epithelia of the apical layer and not more widespread throughout the epithelium. The opposite could also be argued, such that *Piezo1* expression may be limited given the repetitive biomechanical demands to murine VF mucosa. Recent work may support the latter notion, indicating transduction limits of *Piezo* channels, which turn out to be poor discriminators and inefficient transducers of continuous high-frequency stimulations in other organs/cell types [98]. It will be of interest for future work to investigate *Piezo1* in human VF mucosa to identify species similarities/comparisons and functional roles for translational impact.

VF epithelia are short-lived and undergo constant turnover and renewal throughout life. In addition, epithelial cells are the initial cell type in the VF to encounter toxic exposure, which may make them principal modulators of VF disease development [15, 17]. We utilized the chemical toxin naphthalene, commonly found in a variety of pollutants/irritants (wood smoke, tar, asphalt, fossil fuels), and inhaled carcinogens (tobacco smoke) [48, 52, 99], to injure VF mucosa which specifically targets damage to epithelial cells via its effect on the cytochrome P450 system [48–54]. To assess *Piezo1*-mediated repair pathways, adult 6wk control and mutant VF epithelium underwent injury for analysis at 1-, 3-, 7- and 14-dpi. NAPH exposure to WT VF epithelium resulted in appreciated injury at 3 dpi, characterized by epithelial hyperplasia along with increased EdU^+^ cell proliferation and H2AX^+^ DNA damage. We also demonstrated that NAPH injury resulted in P63^+^ basal cell compartment expansion with transient sensitivity to SCGB1A1^+^ epithelia, denoted by near complete ablation at 3 dpi with population rebound by 7 dpi. Our data confirm previous molecular findings regarding NAPH effect on airway epithelium, albeit, with varying rates of cellular injury and repair [48, 50, 65].

We then hypothesized that injury to VF mutant epithelium would mimic an elevated mechanical environment, ultimately surpassing *Piezo1* channel thresholds, for activation of aberrant downstream pathways following *Piezo1* loss. The basis of this assumption was that *Piezo1*- expressing VF epithelial ion channels are likely of the high-threshold type that only respond to intense mechanical stimuli and/or injury [100]. Consistent with our hypothesis, NAPH-exposure in *Piezo1* mutant epithelium resulted in increasingly severe VF injury at 3 dpi. Specifically, *Shh*^*cre/*+^*;Piezo1*^*cko*^*,Piezo2*^*loxP/*+^ VF epithelial defects were characterized by loss of stratification, disorganized/large squamous cells with polymorphous nuclei, irregular nuclear borders, and large amounts of cytoplasm with indistinct cell borders. Consistent with other work in rodent VF injury models [25, 85–89], epithelium regeneration and structural restoration was appreciated by 7 dpi in WT control and mutant colonies. Previous in vitro work has shown the role of mechanosensitive channels as primary transducers in force transmission to the nucleus [101], with shear stress-induced nuclear shrinkage through activation of *Piezo1* channels in epithelial cells [102]. Our in vivo data present related findings, such that *Piezo1* epithelial loss appeared to modulate and enlarge nuclear size, however, whether this results from lack of Ca^2+^ signaling and/or direct mechanical interactions of actin with the nuclear membrane remains unknown.

Next, we turned our attention to investigating YAP expression given the importance of Hippo/YAP pathway for VF epithelium morphogenesis [68], and previous work implicating *Piezo1* as an upstream component for YAP transduction pathways [69, 75]. *Shh*^*cre/*+^*;Piezo1/2*^*loxP/*+^ and *Shh*^*cre/*+^*;Piezo1*^*cko*^*,Piezo2*^*loxP/*+^ mutant VF epithelium revealed a clear shift from cytoplasmic-to-nuclear YAP expression compared to control following NAPH injury. In addition, increased YAP^+^P63^+^ cell co-localization was exhibited following *Piezo1* ablation compared to heterozygote epithelium. qPCR revealed significantly increased *p63* mRNA abundance in NAPH-exposed *Shh*^*cre/*+^*;Piezo1*^*cko*^*,Piezo2*^*loxP/*+^ mutant compared to heterozygote and control epithelium, albeit, no differences were noted in either *Yap1* and/or *Yap2* mRNA transcripts. These effects, following *Piezo1* ablation, suggest posttranslational modification of YAP facilitating subcellular localization, which may have a direct effect on *p63* resulting in epithelial cell proliferation and stratification defects. These data support prior work that has demonstrated genetic interaction of *p63* and *Yap* in airway epithelium [74]; with other known roles for epithelial proliferation and stratification events in skin [70–73].

To assess downstream cell signaling events for SSE remodeling, we stained cytokeratin differentiation markers K13 and K14, as well as K17—a marker known to be upregulated during wounding [77] and DNA damage [76]. K14 has been shown preferentially located to basal VF epithelia with diffusion across all cell layers [56, 64], believed to play a pivotal role in cell shape and resistance to mechanical trauma in mouse epidermis [78]. Our data confirm previous work with increased K14 expression across all layers of WT VF epithelium at 3 dpi, extending into distal trachea to K14^+^ basal cells and submucosal compartments. In addition, K13, a marker of differentiated SSE, was overexpressed with noted ectopic positivity to non-squamous cells at the squamociliary junction of the subglottis. K17, not normally expressed in healthy VF epithelium, exhibited no expression to either WT uninjured and/or injured epithelium. Together, data suggest predilection for unique cellular changes to the laryngeal subglottic region following insult supporting vulnerability at epithelium transition zones in the larynx [103–105]. Further work is warranted to establish whether these cells hold any particular significance for VF barrier function and immunomodulation.

Elevated expression of K17 has also been shown to play a role in head and neck SCC associated with decreased survival rates [79–81], in addition to its role in mouse papillomavirus [57]. Interestingly, *Shh*^*cre/*+^*;Piezo1/2*^*loxP/*+^ mutant epithelium exhibited ectopic K17 focal emergence to the squamociliary junction in uninjured corn oil controls, extending to SSE of the VF and laryngeal surface of the epiglottis following NAPH injury. Emergence of K17 with analogous loss of K13 has been found associated with more invasive malignant disease in oral SCC [79]. NAPH-exposed *Shh*^*cre/*+^; *Piezo1/2*^*loxP/*+^ and *Shh*^*cre/*+^; *Piezo1*^*cko*^, *Piezo2*^*loxP/*+^ mutant epithelium revealed what seemed to be reciprocal expression patterns for K13/K17, such that ectopic K17 expression to VF epithelial cells led to an inverse loss of K13 expression. qPCR analysis confirmed significantly increased K17 mRNA fold change comparing *Piezo1* mutant epithelium to control, albeit, no K13 differences were exhibited. Researchers have hypothesized that K17 may serve as an early marker for epithelial transformation [81], therefore, we stained for N-cadherin and SNAIL1, known markers for EMT during gastrulation and tumorigenesis [106, 107]. Results were negative, ruling out the possibility of EMT to VF epithelium following *Piezo1* loss with combined NAPH injury. While our data suggest possible links between *Piezo1* signaling in K13-expressing VF apical epithelia and aberrant K17 expression, downstream pathways remain unclear with further work warranted.

Lastly, we were interested in long-term remodeling effects to VF barrier integrity following *Piezo1* loss. SSE provides the VF with a protective barrier against mechanical, chemical and biological insult. Two well-known proteins important for maintaining cell–cell adhesion in SSE include E-cadherin and ZO1, markers of adheren and tight junctions, respectively. By 3 dpi, WT NAPH-injured controls demonstrated excessive E-cadherin and ZO1 proteins, with mislocalized ZO1 penetrating to basal stratum compared to corn oil controls. By 3 dpi, NAPH-exposed *Shh*^*cre/*+^*;Piezo1*^*cko*^*,Piezo2*^*loxP/*+^ mutant epithelium exhibited disrupted E-cadherin and ZO1 proteins. Interestingly, fragmented ZO1 expression was displayed along the apical edge of corn oil uninjured *Shh*^*cre/*+^*;Piezo1*^*cko*^*,Piezo2*^*loxP/*+^ mutant epithelium. By 14 dpi, *Shh*^*cre/*+^*;Piezo1*^*cko*^*,Piezo2*^*loxP/*+^ mutant epithelium continued to exhibit compromise to barrier integrity by fragmented ZO1 expression in both corn oil-uninjured and NAPH-injured treatment conditions. This suggests that despite structural epithelial restoration by 7 dpi, barrier integrity may be delayed up to 14 days or longer following injury within an epithelium that lacks *Piezo1* epithelial cell ion channels. Ongoing compromise of regenerated epithelium implies that VFs may remain at risk for recurrent damage, pathogen invasion and remodeling. More long-term studies for *Piezo1* importance to VF barrier function following injury are required in both animal and in vitro human air–liquid interface model systems for comparative analysis and translational impact.

A few limitations of the present work need to be addressed. First, while experiments explored mechanistic insight by which *Piezo1* functions to regulate VF epithelium, inherent genetic redundancy of *Piezo* mechanoreceptors to tissue- and cell-function exist [108, 109]. This effect is assumed across many upstream transduction mechanisms [110], enacting safeguards for continued cell function and repair strategies. We accounted for this scenario by creating double mutants for *Piezo1* and *Piezo2* null alleles from the *shh* promotor. This, however, both extended our study for mutant tissue collection and limited our total number for investigative analyses. We found *Piezo1,* only, to be important for VF epithelium. To this end, we did not appreciate a phenotype without concurrent injury, thus, we cannot rule out that this may be due to inefficient complete genetic *Piezo1* deletion. Residual *Piezo1* expression may have been sufficient to prevent aberrant epithelial signaling, albeit, with injury this threshold may have been surpassed leading to our phenotype. Another limitation was that NAPH-induced injury was not 100% reproducible. Visual examination of WT histologic samples exhibited a 60% success rate for VF injury at 3 dpi (*n* = 16) with a 100% survival rate by 14 dpi (*n* = 5). Evidence suggests dose concentration [111], time of dose (circadian rhythms) [112], murine background strain [113], sex [51] and likely host immune response all contribute to variability in injury uptake and severity. Prior research in the lung and tracheal airways has utilized intraperitoneal dose concentrations ranging from 200 to 300 mg/kg [48–51, 65, 66, 111]. We chose a higher dose of 275 mg/kg given less concentrated secretory cells in the larynx and work suggesting higher doses are required for injury in proximal lung regions. For our intent purposes, we were able to use the proximal tracheal airway as an internal control for injury uptake at earlier timepoint analyses (1 & 3 dpi) due to the large effect on concentrated club cells in tracheal epithelium, especially at this higher dose. Furthermore, on account of our initial data we mainly focused our efforts on characterizing injury at day 3 following NAPH exposure. A thorough examination of all time points across wound healing phases is necessary to elucidate VF remodeling events in a murine model of regeneration.

**Fig. 10:**
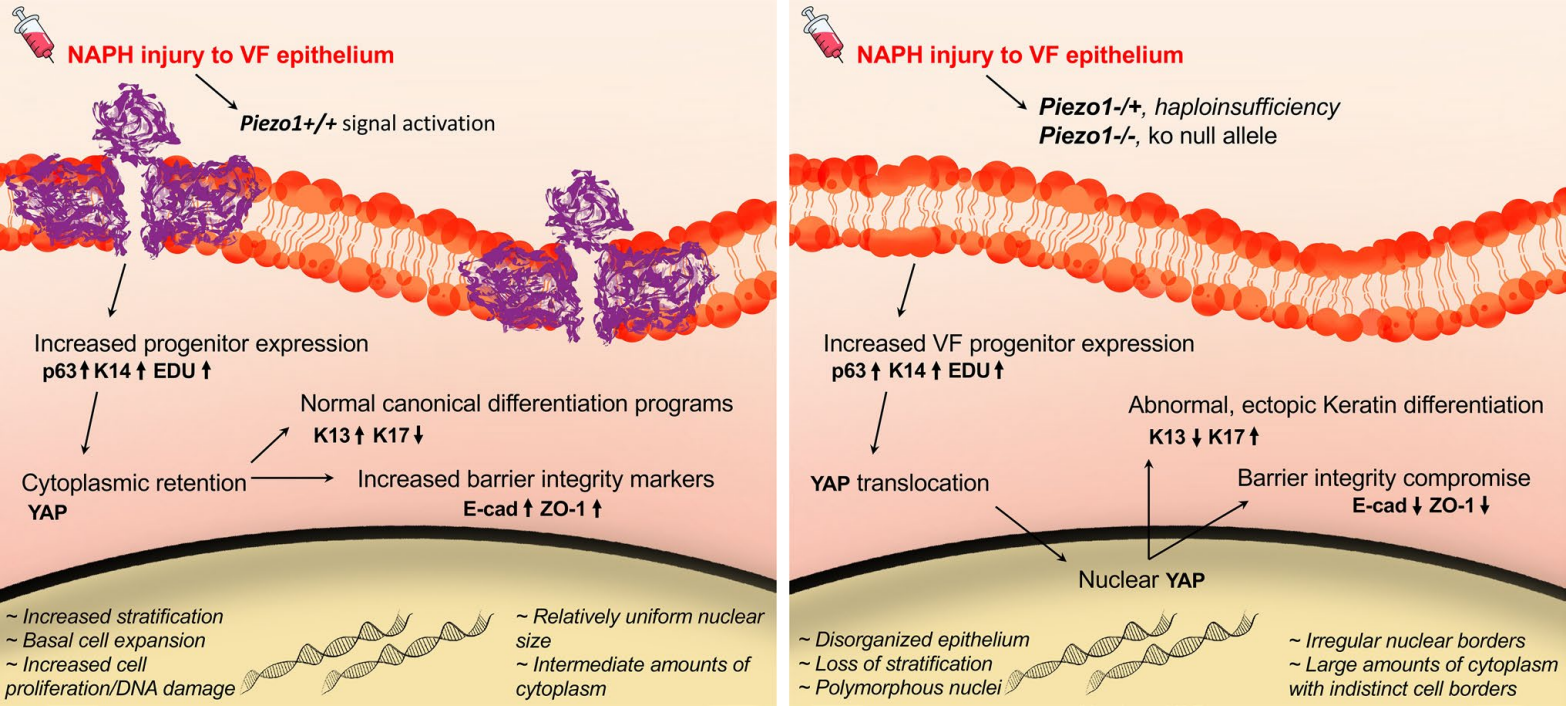
Schematic illustration of proposed function for *Piezo1* regulation in VF epithelial remodeling

Taken together, findings from this investigation improve our understanding of acute VF wound healing in the context of *Piezo1* epithelial function and form the basis for an in vivo methodology to study VF responses to NAPH injury. In vitro model systems with *Piezo1* gain-of- and loss-of-function are warranted to further clarify and refine the mechanism of *Piezo1* action in VF regeneration and remodeling following NAPH exposure. In addition, use of pharmacological agents, such as *Piezo1* agonists (Yoda1, Jedi1/2) and/or antagonists (Dooku1, Gadolinium, GsMTx4) would provide evidence for a potential use as therapeutic interventions. Recent interest has been given to the emerging role of *Piezo1* in meningeal nociception leading to migraine headache, [114, 115] with speculation into roles for modulating/mitigating VF mucosal injury. We conclude that *Piezo1* mechanosensitive ion channels play a crucial role in VF regeneration and remodeling, likely through regulating epithelial self-renewal via effects on *p63* transcription and YAP subcellular localization—altering cytokeratin differentiation (Fig. 10).

## Supplementary Information

Below is the link to the electronic supplementary material.

Supplementary file1 (PDF 15664 KB)

## Data Availability

The datasets generated during and/or analyzed during the current study are available from the corresponding author on reasonable request.

## References

[1] Sanders I, Mu L (1998). Anatomy of the human internal superior laryngeal nerve. Anat Rec.

[2] Bradley RM (2000). Sensory receptors of the larynx. Am J Med.

[3] Jette ME, Clary MS, Prager JD, Finger TE (2019). Chemical receptors of the arytenoid: a comparison of human and mouse. Laryngoscope.

[4] Sant'Ambrogio G, Mathew OP, Sant'Ambrogio FB, Fisher JT (1985). Laryngeal cold receptors. Respir Physiol.

[5] Bianconi R, Molinari G (1962). Electroneurographic evidence of muscle spindles and other sensory endings in the intrinsic laryngeal muscles of the cat. Acta Otolaryngol.

[6] Hamamoto T, Takumida M, Hirakawa K, Tatsukawa T, Ishibashi T (2009). Localization of transient receptor potential vanilloid (TRPV) in the human larynx. Acta Otolaryngol.

[7] Yamamoto Y, Taniguchi K (2005). Immunolocalization of VR1 and VRL1 in rat larynx. Auton Neurosci.

[8] Hamamoto T, Takumida M, Hirakawa K, Takeno S, Tatsukawa T (2008). Localization of transient receptor potential channel vanilloid subfamilies in the mouse larynx. Acta Otolaryngol.

[9] Uno T, Koike S, Bamba H, Hirota R, Hisa Y (2004). Capsaicin receptor expression in rat laryngeal innervation. Ann Otol Rhinol Laryngol.

[10] Andreatta RD, Mann EA, Poletto CJ, Ludlow CL (2002). Mucosal afferents mediate laryngeal adductor responses in the cat. J Appl Physiol.

[11] Davis PJ, Nail BS (1987). Quantitative analysis of laryngeal mechanosensitivity in the cat and rabbit. J Physiol.

[12] Hammer MJ, Krueger MA (2014). Voice-related modulation of mechanosensory detection thresholds in the human larynx. Exp Brain Res.

[13] Prescott SL, Umans BD, Williams EK, Brust RD, Liberles SD (2020). An airway protection program revealed by sweeping genetic control of vagal afferents. Cell.

[14] Ludlow CL (2015). Laryngeal reflexes: physiology, technique, and clinical use. J Clin Neurophysiol.

[15] Levendoski EE, Leydon C, Thibeault SL (2014). Vocal fold epithelial barrier in health and injury: a research review. J Speech Lang Hear Res.

[16] Erickson E, Sivasankar M (2010). Simulated reflux decreases vocal fold epithelial barrier resistance. Laryngoscope.

[17] Erickson-DiRenzo E, Easwaran M, Martinez JD, Dewan K, Sung CK (2021). Mainstream cigarette smoke impacts the mouse vocal fold epithelium and mucus barrier. Laryngoscope.

[18] Titze IR (1994). Mechanical stress in phonation. J Voice.

[19] Novaleski CK, Kimball EE, Mizuta M, Rousseau B (2016). Acute exposure to vibration is an apoptosis-inducing stimulus in the vocal fold epithelium. Tissue Cell.

[20] Khosla S, Murugappan S, Gutmark E (2008). What can vortices tell us about vocal fold vibration and voice production. Curr Opin Otolaryngol Head Neck Surg.

[21] Khosla S, Murugappan S, Paniello R, Ying J, Gutmark E (2009). Role of vortices in voice production: normal versus asymmetric tension. Laryngoscope.

[22] Ling C, Yamashita M, Waselchuk EA, Raasch JL, Bless DM, Welham NV (2010). Alteration in cellular morphology, density and distribution in rat vocal fold mucosa following injury. Wound Repair Regen.

[23] Gurtner GC, Werner S, Barrandon Y, Longaker MT (2008). Wound repair and regeneration. Nature.

[24] Branski RC, Verdolini K, Sandulache V, Rosen CA, Hebda PA (2006). Vocal fold wound healing: a review for clinicians. J Voice.

[25] Yamashita M, Bless DM, Welham NV (2010). Morphological and extracellular matrix changes following vocal fold injury in mice. Cells Tissues Organs.

[26] Lungova V, Verheyden JM, Sun X, Thibeault SL (2018). beta-Catenin signaling is essential for mammalian larynx recanalization and the establishment of vocal fold progenitor cells. Development.

[27] Dowdall JR, Sadow PM, Hartnick C, Vinarsky V, Mou H, Zhao R (2015). Identification of distinct layers within the stratified squamous epithelium of the adult human true vocal fold. Laryngoscope.

[28] Bailly L, Cochereau T, Orgeas L, Henrich-Bernardoni N, Rolland-du-Roscoat S, McLeer-Florin A (2018). 3D multiscale imaging of human vocal folds using synchrotron X-ray microtomography in phase retrieval mode. Sci Rep.

[29] Coste B, Mathur J, Schmidt M, Earley TJ, Ranade S, Petrus MJ (2010). Piezo1 and Piezo2 are essential components of distinct mechanically activated cation channels. Science.

[30] Syeda R, Florendo MN, Cox CD, Kefauver JM, Santos JS, Martinac B (2016). Piezo1 channels are inherently mechanosensitive. Cell Rep.

[31] Gudipaty SA, Lindblom J, Loftus PD, Redd MJ, Edes K, Davey CF (2017). Mechanical stretch triggers rapid epithelial cell division through Piezo1. Nature.

[32] Eisenhoffer GT, Loftus PD, Yoshigi M, Otsuna H, Chien CB, Morcos PA (2012). Crowding induces live cell extrusion to maintain homeostatic cell numbers in epithelia. Nature.

[33] Jiang Y, Yang X, Jiang J, Xiao B (2021). Structural designs and mechanogating mechanisms of the mechanosensitive piezo channels. Trends Biochem Sci.

[34] Huo L, Gao Y, Zhang D, Wang S, Han Y, Men H (2021). Piezo2 channel in nodose ganglia neurons is essential in controlling hypertension in a pathway regulated directly by Nedd4-2. Pharmacol Res.

[35] Shin SM, Moehring F, Itson-Zoske B, Fan F, Stucky CL, Hogan QH (2021). Piezo2 mechanosensitive ion channel is located to sensory neurons and non-neuronal cells in rat peripheral sensory pathway: implications in pain. bioRxiv..

[36] Nonomura K, Woo SH, Chang RB, Gillich A, Qiu Z, Francisco AG (2017). Piezo2 senses airway stretch and mediates lung inflation-induced apnoea. Nature.

[37] Assaraf E, Blecher R, Heinemann-Yerushalmi L, Krief S, Carmel Vinestock R, Biton IE (2020). Piezo2 expressed in proprioceptive neurons is essential for skeletal integrity. Nat Commun.

[38] Alcaino C, Knutson KR, Treichel AJ, Yildiz G, Strege PR, Linden DR (2018). A population of gut epithelial enterochromaffin cells is mechanosensitive and requires Piezo2 to convert force into serotonin release. Proc Natl Acad Sci U S A.

[39] Woo SH, Ranade S, Weyer AD, Dubin AE, Baba Y, Qiu Z (2014). Piezo2 is required for Merkel-cell mechanotransduction. Nature.

[40] Maksimovic S, Nakatani M, Baba Y, Nelson AM, Marshall KL, Wellnitz SA (2014). Epidermal Merkel cells are mechanosensory cells that tune mammalian touch receptors. Nature.

[41] Woo S-H, Lukacs V, De Nooij JC, Zaytseva D, Criddle CR, Francisco A (2015). Piezo2 is the principal mechanotransduction channel for proprioception. Nat Neurosci.

[42] Kim SE, Coste B, Chadha A, Cook B, Patapoutian A (2012). The role of Drosophila Piezo in mechanical nociception. Nature.

[43] Sinclair CF, Tellez MJ, Ulkatan S (2018). Human laryngeal sensory receptor mapping illuminates the mechanisms of laryngeal adductor reflex control. Laryngoscope.

[44] Aviv JE (1997). Sensory discrimination in the larynx and hypopharynx. Otolaryngol Head Neck Surg.

[45] Aviv JE, Martin JH, Keen MS, Debell M, Blitzer A (1993). Air pulse quantification of supraglottic and pharyngeal sensation: a new technique. Ann Otol Rhinol Laryngol.

[46] Canning BJ, Mazzone SB, Meeker SN, Mori N, Reynolds SM, Undem BJ (2004). Identification of the tracheal and laryngeal afferent neurones mediating cough in anaesthetized guinea-pigs. J Physiol.

[47] Prescott SL, Liberles SD (2022). Internal senses of the vagus nerve. Neuron.

[48] Karagiannis TC, Li X, Tang MM, Orlowski C, El-Osta A, Tang ML (2012). Molecular model of naphthalene-induced DNA damage in the murine lung. Hum Exp Toxicol.

[49] Poulsen TT, Naizhen X, Poulsen HS, Linnoila RI (2008). Acute damage by naphthalene triggers expression of the neuroendocrine marker PGP9.5 in airway epithelial cells. Toxicol Lett.

[50] Hsu HS, Liu CC, Lin JH, Hsu TW, Su K, Hung SC (2014). Repair of naphthalene-induced acute tracheal injury by basal cells depends on beta-catenin. J Thorac Cardiovasc Surg.

[51] Van Winkle LS, Gunderson AD, Shimizu JA, Baker GL, Brown CD (2002). Gender differences in naphthalene metabolism and naphthalene-induced acute lung injury. Am J Physiol Lung Cell Mol Physiol.

[52] Rokicki W, Rokicki M, Wojtacha J, Dzeljijli A (2016). The role and importance of club cells (Clara cells) in the pathogenesis of some respiratory diseases. Kardiochir Torakochirurgia Pol.

[53] Hong KU, Reynolds SD, Watkins S, Fuchs E, Stripp BR (2004). Basal cells are a multipotent progenitor capable of renewing the bronchial epithelium. Am J Pathol.

[54] Ghosh M, Brechbuhl HM, Smith RW, Li B, Hicks DA, Titchner T (2011). Context-dependent differentiation of multipotential keratin 14-expressing tracheal basal cells. Am J Respir Cell Mol Biol.

[55] Council NR (2010) Guide for the care and use of laboratory animals

[56] Lungova V, Verheyden JM, Herriges J, Sun X, Thibeault SL (2015). Ontogeny of the mouse vocal fold epithelium. Dev Biol.

[57] Wang W, Uberoi A, Spurgeon M, Gronski E, Majerciak V, Lobanov A (2020). Stress keratin 17 enhances papillomavirus infection-induced disease by downregulating T cell recruitment. PLoS Pathog.

[58] Nyman PE, Buehler D, Lambert PF (2018). Loss of function of canonical notch signaling drives head and neck carcinogenesis. Clin Cancer Res.

[59] Martinez JD, Easwaran M, Ramirez D, Erickson-DiRenzo E (2022). Effects of electronic (E)-cigarette vapor and cigarette smoke in cultured vocal fold fibroblasts. Laryngoscope.

[60] Ling C, Li Q, Brown ME, Kishimoto Y, Toya Y, Devine EE (2015). Bioengineered vocal fold mucosa for voice restoration. Sci Transl Med..

[61] Jiang J, Lin E, Hanson DG (2000). Vocal fold physiology. Otolaryngol Clin N Am.

[62] Benninger MS, Alessi D, Archer S, Bastian R, Ford C, Koufman J (1996). Vocal fold scarring: current concepts and management. Otolaryngol Head Neck Surg.

[63] Sinclair CF, Tellez MJ, Tapia OR, Ulkatan S (2017). Contralateral R1 and R2 components of the laryngeal adductor reflex in humans under general anesthesia. Laryngoscope.

[64] Lungova V, Chen X, Wang Z, Kendziorski C, Thibeault SL (2019). Human induced pluripotent stem cell-derived vocal fold mucosa mimics development and responses to smoke exposure. Nat Commun.

[65] Fang S, Zhang S, Dai H, Hu X, Li C, Xing Y (2019). The role of pulmonary mesenchymal cells in airway epithelium regeneration during injury repair. Stem Cell Res Ther.

[66] Kovalchuk N, Zhang QY, Van Winkle L, Ding X (2020). Contribution of pulmonary CYP-mediated bioactivation of naphthalene to airway epithelial injury in the lung. Toxicol Sci.

[67] Mou H, Vinarsky V, Tata PR, Brazauskas K, Choi SH, Crooke AK (2016). Dual SMAD signaling inhibition enables long-term expansion of diverse epithelial basal cells. Cell Stem Cell.

[68] Mohad V, Lungova V, Verheyden J, Thibeault SL (2021). Inactivation of Lats1 and Lats2 highlights the role of hippo pathway effector YAP in larynx and vocal fold epithelium morphogenesis. Dev Biol.

[69] Zhou T, Gao B, Fan Y, Liu Y, Feng S, Cong Q (2020). Piezo1/2 mediate mechanotransduction essential for bone formation through concerted activation of NFAT-YAP1-ss-catenin. Elife.

[70] Schlegelmilch K, Mohseni M, Kirak O, Pruszak J, Rodriguez JR, Zhou D (2011). Yap1 acts downstream of α-catenin to control epidermal proliferation. Cell.

[71] Zhang H, Pasolli HA, Fuchs E (2011). Yes-associated protein (YAP) transcriptional coactivator functions in balancing growth and differentiation in skin. Proc Natl Acad Sci U S A.

[72] Romano R-A, Smalley K, Magraw C, Serna VA, Kurita T, Raghavan S (2012). Δ Np63 knockout mice reveal its indispensable role as a master regulator of epithelial development and differentiation. Development.

[73] Koster MI, Kim S, Mills AA, DeMayo FJ, Roop DR (2004). p63 is the molecular switch for initiation of an epithelial stratification program. Genes Dev.

[74] Zhao R, Fallon TR, Saladi SV, Pardo-Saganta A, Villoria J, Mou H (2014). Yap tunes airway epithelial size and architecture by regulating the identity, maintenance, and self-renewal of stem cells. Dev Cell.

[75] Duchemin AL, Vignes H, Vermot J (2019). Mechanically activated piezo channels modulate outflow tract valve development through the Yap1 and Klf2-Notch signaling axis. Elife.

[76] Nair RR, Hsu J, Jacob JT, Pineda CM, Hobbs RP, Coulombe PA (2021). A role for keratin 17 during DNA damage response and tumor initiation. Proc Natl Acad Sci U S A.

[77] Zhang X, Yin M, Zhang LJ (2019). Keratin 6, 16 and 17-critical barrier alarmin molecules in skin wounds and psoriasis. Cells.

[78] Troy TC, Turksen K (1999). In vitro characteristics of early epidermal progenitors isolated from keratin 14 (K14)-deficient mice: insights into the role of keratin 17 in mouse keratinocytes. J Cell Physiol.

[79] Mikami T, Cheng J, Maruyama S, Kobayashi T, Funayama A, Yamazaki M (2011). Emergence of keratin 17 vs loss of keratin 13: their reciprocal immunohistochemical profiles in oral carcinoma in situ. Oral Oncol.

[80] Regenbogen E, Mo M, Romeiser J, Shroyer ALW, Escobar-Hoyos LF, Burke S (2018). Elevated expression of keratin 17 in oropharyngeal squamous cell carcinoma is associated with decreased survival. Head Neck.

[81] Cohen-Kerem R, Rahat MA, Madah W, Greenberg E, Sabo E, Elmalah I (2004). Cytokeratin-17 as a potential marker for squamous cell carcinoma of the larynx. Ann Otol Rhinol Laryngol.

[82] Lim J, Thiery JP (2012). Epithelial-mesenchymal transitions: insights from development. Development.

[83] Nieto MA, Huang RY, Jackson RA, Thiery JP (2016). Emt: 2016. Cell.

[84] Thiery JP, Acloque H, Huang RY, Nieto MA (2009). Epithelial-mesenchymal transitions in development and disease. Cell.

[85] Yamashita M, Bless DM, Welham NV (2009). Surgical method to create vocal fold injuries in mice. Ann Otol Rhinol Laryngol.

[86] Ling C, Raasch JL, Welham NV (2011). E-cadherin and transglutaminase-1 epithelial barrier restoration precedes type IV collagen basement membrane reconstruction following vocal fold mucosal injury. Cells Tissues Organs.

[87] Tateya T, Tateya I, Sohn JH, Bless DM (2006). Histological study of acute vocal fold injury in a rat model. Ann Otol Rhinol Laryngol.

[88] Leydon C, Imaizumi M, Yang D, Thibeault SL, Fried MP (2014). Structural and functional vocal fold epithelial integrity following injury. Laryngoscope.

[89] Suzuki R, Katsuno T, Kishimoto Y, Nakamura R, Mizuta M, Suehiro A (2018). Process of tight junction recovery in the injured vocal fold epithelium: morphological and paracellular permeability analysis. Laryngoscope.

[90] Sanders I, Wu BL, Mu L, Li Y, Biller HF (1993). The innervation of the human larynx. Arch Otolaryngol Head Neck Surg.

[91] Ruoppolo G, Schettino I, Biasiotta A, Roma R, Greco A, Soldo P (2015). Afferent nerve ending density in the human laryngeal mucosa: potential implications on endoscopic evaluation of laryngeal sensitivity. Dysphagia.

[92] Roberts LH (1975). Evidence for the laryngeal source of ultrasonic and audible cries of rodents. J Zool.

[93] Roberts LH (1975). The rodent ultrasound production mechanism. Ultrasonics.

[94] Pasch B, Tokuda IT, Riede T (2017). Grasshopper mice employ distinct vocal production mechanisms in different social contexts. Proc Biol Sci.

[95] Sangiamo DT, Warren MR, Neunuebel JP (2020). Ultrasonic signals associated with different types of social behavior of mice. Nat Neurosci.

[96] Riede T, Borgard HL, Pasch B (2017). Laryngeal airway reconstruction indicates that rodent ultrasonic vocalizations are produced by an edge-tone mechanism. Roy Soc Open Sci.

[97] Mahrt E, Agarwal A, Perkel D, Portfors C, Elemans CP (2016). Mice produce ultrasonic vocalizations by intra-laryngeal planar impinging jets. Curr Biol.

[98] Lewis AH, Cui AF, McDonald MF, Grandl J (2017). Transduction of repetitive mechanical stimuli by Piezo1 and Piezo2 ion channels. Cell Rep.

[99] Preuss R, Angerer J, Drexler H (2003). Naphthalene—an environmental and occupational toxicant. Int Arch Occup Environ Health.

[100] Olson W, Dong P, Fleming M, Luo W (2016). The specification and wiring of mammalian cutaneous low-threshold mechanoreceptors. Wiley Interdiscip Rev Dev Biol.

[101] Jetta D, Bahrani Fard MR, Sachs F, Munechika K, Hua SZ (2021). Adherent cell remodeling on micropatterns is modulated by Piezo1 channels. Sci Rep.

[102] Jetta D, Gottlieb PA, Verma D, Sachs F, Hua SZ (2019). Shear stress-induced nuclear shrinkage through activation of Piezo1 channels in epithelial cells. J Cell Sci.

[103] Sagartz JW, Madarasz AJ, Forsell MA, Burger GT, Ayres PH, Coggins CR (1992). Histological sectioning of the rodent larynx for inhalation toxicity testing. Toxicol Pathol.

[104] Lewis DJ (1991). Morphological assessment of pathological changes within the rat larynx. Toxicol Pathol.

[105] Kashima H, Mounts P, Leventhal B, Hruban RH (1993). Sites of predilection in recurrent respiratory papillomatosis. Ann Otol Rhinol Laryngol.

[106] Loh CY, Chai JY, Tang TF, Wong WF, Sethi G, Shanmugam MK (2019). The E-Cadherin and N-Cadherin switch in epithelial-to-mesenchymal transition: signaling, therapeutic implications, and challenges. Cells.

[107] Scheibner K, Schirge S, Burtscher I, Buttner M, Sterr M, Yang D (2021). Epithelial cell plasticity drives endoderm formation during gastrulation. Nat Cell Biol.

[108] Zeng WZ, Marshall KL, Min S, Daou I, Chapleau MW, Abboud FM (2018). PIEZOs mediate neuronal sensing of blood pressure and the baroreceptor reflex. Science.

[109] Zhang M, Wang Y, Geng J, Zhou S, Xiao B (2019). Mechanically activated Piezo channels mediate touch and suppress acute mechanical pain response in mice. Cell Rep.

[110] Kafri R, Springer M, Pilpel Y (2009). Genetic redundancy: new tricks for old genes. Cell.

[111] West JA, Pakehham G, Morin D, Fleschner CA, Buckpitt AR, Plopper CG (2001). Inhaled naphthalene causes dose dependent Clara cell cytotoxicity in mice but not in rats. Toxicol Appl Pharmacol.

[112] Gibbs JE, Beesley S, Plumb J, Singh D, Farrow S, Ray DW (2009). Circadian timing in the lung; a specific role for bronchiolar epithelial cells. Endocrinology.

[113] Lawson GW, Van Winkle LS, Toskala E, Senior RM, Parks WC, Plopper CG (2002). Mouse strain modulates the role of the ciliated cell in acute tracheobronchial airway injury-distal airways. Am J Pathol.

[114] Della Pietra A, Mikhailov N, Giniatullin R (2020). The emerging role of mechanosensitive Piezo channels in migraine pain. Int J Mol Sci.

[115] Dolgorukova A, Isaeva JE, Verbitskaya E, Lyubashina OA, Giniatullin Rcapital AC, Sokolov AY (2021). Differential effects of the Piezo1 agonist Yoda1 in the trigeminovascular system: an electrophysiological and intravital microscopy study in rats. Exp Neurol.

