# Supplementary material for: Piezo1-expressing vocal fold epithelia modulate remodeling via effects on self-renewal and cytokeratin differentiation
Source: Cell Mol Life Sci. 2022 Nov 14;79(12):591. doi: 10.1007/s00018-022-04622-6 (PMC9663367; doi:10.1007/s00018-022-04622-6)
Supplement: Supplementary file 1 — Supplementary file1 (PDF 15664 KB) [file 18_2022_4622_MOESM1_ESM.pdf]

Table S1. Primers used in this study.

qPCR Primers	Forward 5'- 3'	Reverse 3'-5'	Manufacturer
Piezo1	GCC TAG ATT CAC CTG GCT TC	GCT CTT AAC CAT TGA GCC ATC T	Integrated DNA Technologies
Piezo2	ACC ATG ATC AAG TGG ACT AGA GA	CAG CCA CCT GTC AGA AGA GA	Integrated DNA Technologies
ΔNp63	GGA AAA CAA TGC CCA GAC TC	GAT GGA GAG AGG GCA TCA AA	Integrated DNA Technologies
Yap1	TAC ATA AAC CAT AAG AAC AAG ACC ACA	GCT TCA CTG GAG CAC TCT GA	Integrated DNA Technologies
Yap2	CAT CTT CTG GTC AAA GAT ACT TC	CAG AAT TCA TCA GCG TCT G	Integrated DNA Technologies
K13	TCA TCT CGG TTT GTC ACT GGA	TGA TCT TCT CGT TGC CAG AGA G	Integrated DNA Technologies
K17	Mm00495207_m1	Mm00495207_m1	ThermoFisher
B-actin	TGG AAT CCT GTG GCA TCC ATG AAA C	TAA AAC GCA GCT CAG TAA CAG TCC G	Integrated DNA Technologies
**Mouse Genotype Primers**	**Sequence 5'- 3'**		**Product size**
Cre	Common, GGGACAGCTCACAAGTCCTC	WT Rev, CTCGGCTACGTTGGGAATAA MUT Rev, GGTGCGCTCCTGGACGTA	*Shh* allele - 350 bp WT allele - 200 bp
tdTomato	WT Fwd, AAGGGAGCTGCAGTGGAGTA MUT Fwd, CTGTTCCTGTACGGCATGG	WT Rev, CCGAAAATCTGTGGGAAGTC MUT Rev, GGCATTAAAGCAGCGTATCC	*tdTomato* allele - 200 bp WT allele - 300 bp
Piezo1$^{loxP/loxP}$	Fwd, GCCTAGATTCACCTGGCTTC	Rev, GCTCTTAACCATTGAGCCATCT	*Piezo1* allele - 380 bp WT allele - 188 bp
Piezo2$^{loxP/loxP}$	WT Fwd, ACTTAGATGGGGCAGGTGCT MUT Fwd, ATCTACCACGGGGCTCTCTC	WT Rev, ACTTCCCTACCCACCCATTC MUT Rev, GCCGCTCTAGAACTAGTGGA	*Piezo2* allele - 171 bp WT allele - 109 bp

Table S2. Antibodies used in this study.

Primary Antibodies	Dilution	Supplier	Catalog #	Lot #
Rabbit anti-PIEZO1	1:100	Novus Biologicals	78446	B4, B5, B6
PIEZO1, block peptide	10-to-1	Novus Biologicals	78446PEP	A4
Rabbit anti-PIEZO2	1:200 (TSA)	Novus Biologicals	78624	E, F
PIEZO2, block peptide	10-to-1	Novus Biologicals	78624PEP	A2
Mouse, anti-K13	1:100	ThermoFisher	MA1-35542	VG3031151
Rabbit, anti-K14	1:500	ThermoFisher	RB-9020-P	9020P1202C
Rabbit, anti-K17	1:100	Abcam	ab109725	GR205504-9
Mouse, anti-P63	1:100	Biomedical	CM-163-A	040720A, 080621A, 080621A-2
Rabbit, anti-YAP (total)	1:100 (TSA)	Cell Signaling Technology	14074	4
Mouse, anti-MUC1	1:100	Abcam	ab70475	n/a
Mouse, anti-RFP	1:100	Abcam	ab125244	GR3376997-1
Chicken, anti-UCH-L1/PGP9.5	1:1500	Novus Biologicals	NB110-58872	7185-3
Mouse, anti-phospho-Histone, H2a.X (Ser139)	1:100	EMD Millipore Corp.	05-636	3782118
Rabbit, anti-E-cadheren	1:200	Cell Signaling Technology	3195S	13
Mouse, anti-ZO1	1:200	ThermoFisher	33-9100	UB280528
Rabbit, anti-N-Cadherin (D4R1H)	1:100	Cell Signaling Technology	13116T	4
Rabbit, anti-Snail1 (C15D3)	1:500	Cell Signaling Technology	3879T	14
Rabbit anti-Uteroglobin/SCGB1A1	1:200	Abcam	ab213203	GR3313014-3
**Secondary Antibodies**	**Dilution**	**Supplier**	**Catalog #**	
Alexa Flour 488, Goat anti-Rabbit IgG	1:500	ThermoFisher	A27034	UD2749322
Goat anti-mouse IgG, Cy3™	1:200	Jackson Immuno Research	103-165-155	150532
Goat anti-chicken IgG, Cy3™	1:200	Jackson Immuno Research	115-166-003	144676
**Commercial Kits**				
Alexa Flour 488, Tyramide SuperBoost	n/a	ThermoFisher	B40943	2291406
Click-it™ EdU Alexa Flour™ 488	n/a	ThermoFisher	C10337	2387287
ReliaPrep™ RNA Tissue Miniprep System	n/a	Promega	Z6111	0000507756
GoScript™ Reverse Transcription Mix	n/a	Promega	A2800	0000483840

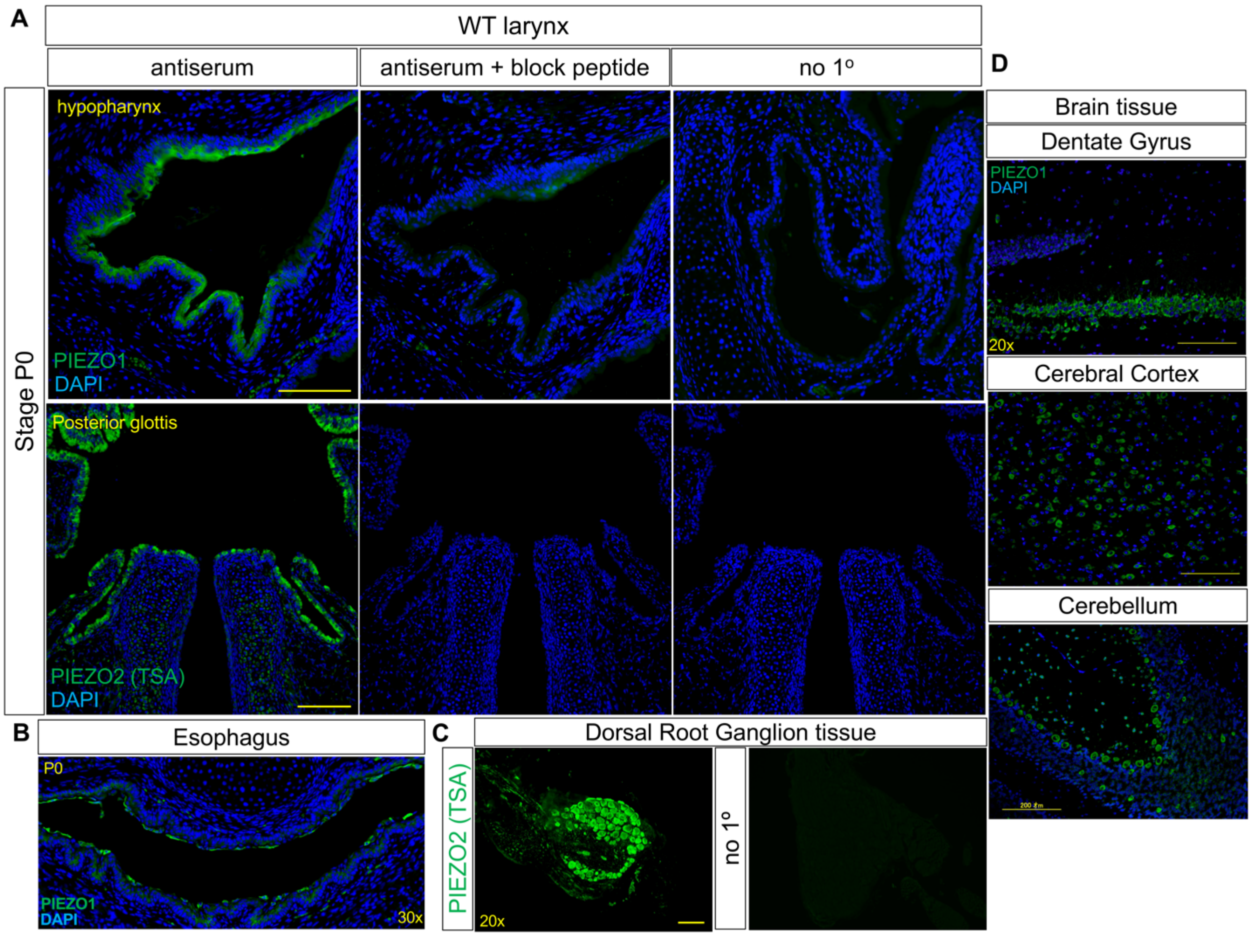

Fig. S1 Positive and negative PIEZO antibody controls. **(a)** IF analysis of serial sections for PIEZO1 (green) and PIEZO2 (green) antibodies exhibit strong expression with antiserum and lack of expression in either antiserum plus blocking peptide and/or when no primary antibody was applied in no cre WT murine Bl6 larynx. **(b)** IF analysis exhibited PIEZO1 expression to apical epithelia of the esophagus at P0 timepoint. **(c)** IF analysis exhibited effective labeling of PIEZO2 to dorsal root ganglion using a tyramide signal amplification kit when antiserum was applied (positive control) and when no primary antiserum was applied (negative control). DAPI is in blue. PIEZO1 images taken at 40X and PIEZO2 images taken at 30X magnification in **(a).** Scale bar represents 100 µm for images in **(a-c)** and 200 µm for images in **(d).** WT: wildtype, P: postnatal, TSA: tyramide signal amplification.

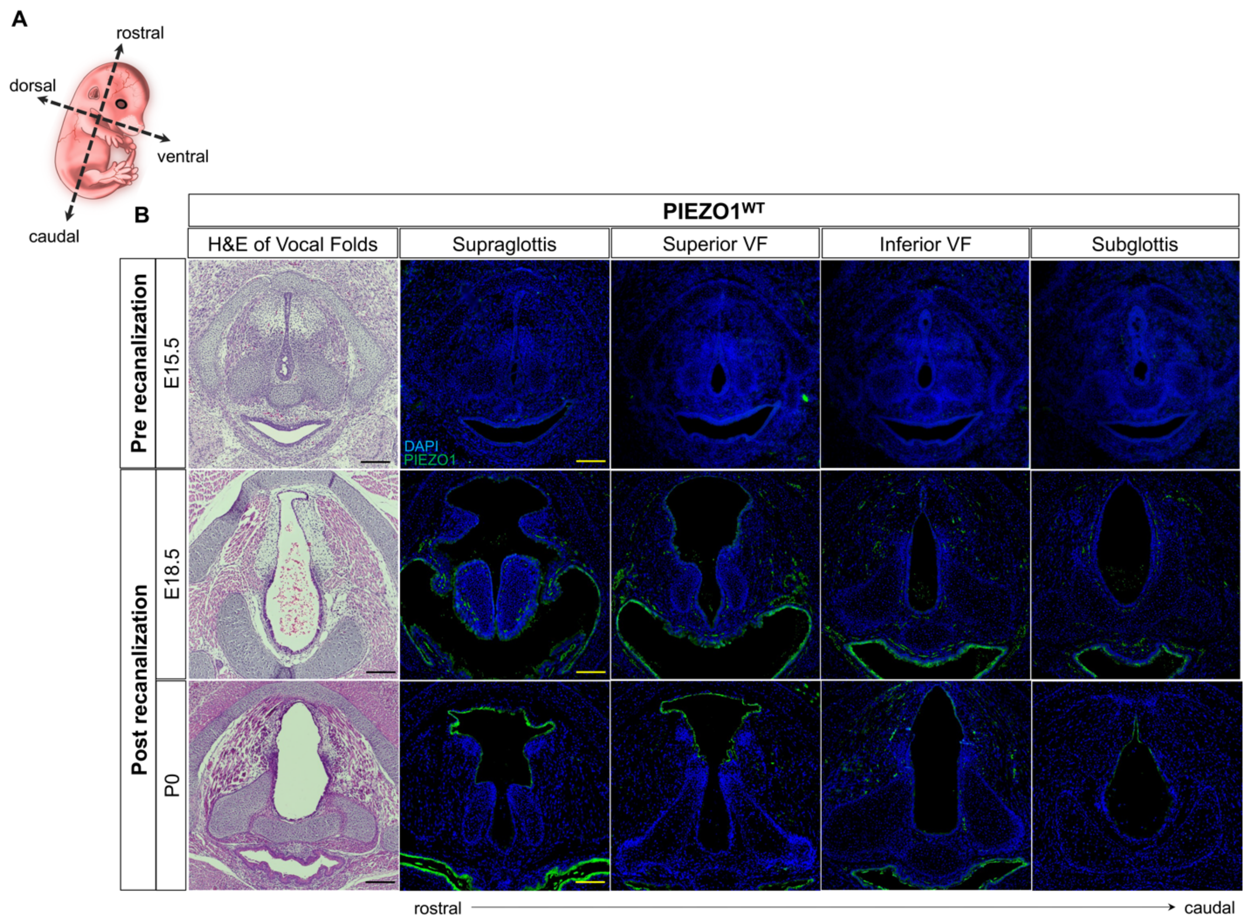

Fig. S2 (a) Schematic illustration of murine anatomic axis. **(b)** Epithelial PIEZO1 *de novo* protein expression coincidences with laryngeal recanalization. H&E-stained transverse sections of larynx at embryonic (E) day 15.5, E18.5 and postnatal (P) day 0 timepoints. Serial sections of PIEZO1 (green) IF protein localization in WT murine Bl6 larynx. Endoderm-derived airway epithelia exhibit selective expression of PIEZO1 to ventral glottis with increased focal expression patterns to stratified, squamous epithelial cells of the esophagus and VF following recanalization at E18.5 and P0 timepoints. No PIEZO1-epithelial cell expression at E15.5 timepoint. DAPI is in blue. All images taken at 20X magnification. Scale bar represents 100 µm. H&E: hematoxylin and eosin, WT: wildtype, VF: vocal fold.

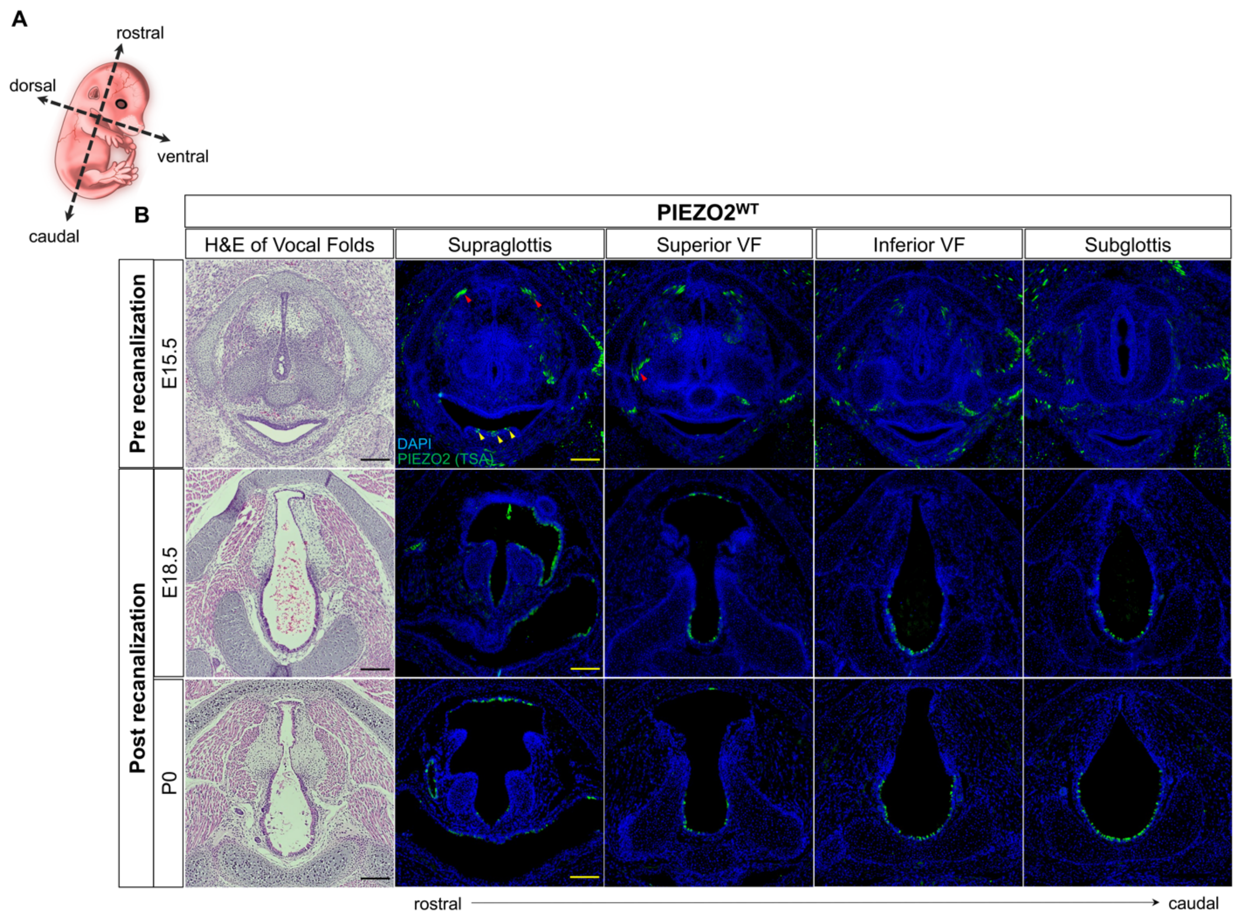

Fig. S3 (a) Schematic illustration of murine anatomic axis. **(b)** Epithelial PIEZO2 *de novo* protein expression coincidences with laryngeal recanalization. H&E-stained transverse sections of larynx at embryonic (E) day 15.5, E18.5 and postnatal (P) day 0 timepoints. Serial sections of PIEZO2 (green) IF protein localization in WT murine Bl6 larynx. Selective expression of PIEZO2 to respiratory, non-squamous epithelial cells following VF recanalization at E18.5 and P0 timepoints. No PIEZO2 expression to airway epithelial cells at E15.5 timepoint. Red arrows indicates PIEZO2 expression to mesenchymal-derived muscle fibers prior to recanalization at E15.5 timepoint. Yellow arrows indicates PIEZO2 expression to endoderm-derived esophageal epithelia prior to recanalization at E15.5. DAPI is in blue. All images taken at 20X magnification. Scale bar represents 100 µm. H&E: hematoxylin and eosin, WT: wildtype, VF: vocal fold, TSA: tyramide signal amplification.

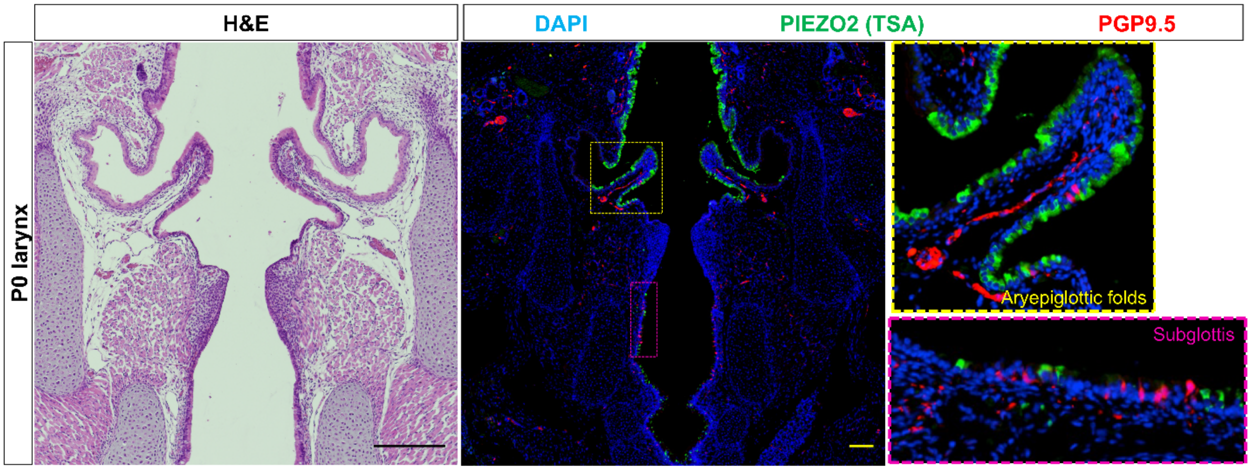

Fig. S4 Nerve endings arborize in supra- and subglottis in close association with PIEZO2 selective expression to P0 larynx. 15X magnification of H&E-stained coronal section of murine larynx. 10X magnification of IF-stained coronal section to exhibit PIEZO2-expressing epithelial cells (green) in close approximation to pan-neuronal marker PGP9.5 innervating nerve fibers (red). Insets are magnified regions from 10X IF; yellow = aryepiglottic folds, purple = subglottis. DAPI is in blue. Scale bar represents 100 µm. H&E: hematoxylin and eosin, IF: immunofluorescence, P0: postnatal day 0, TSA: tyramide signal amplification.

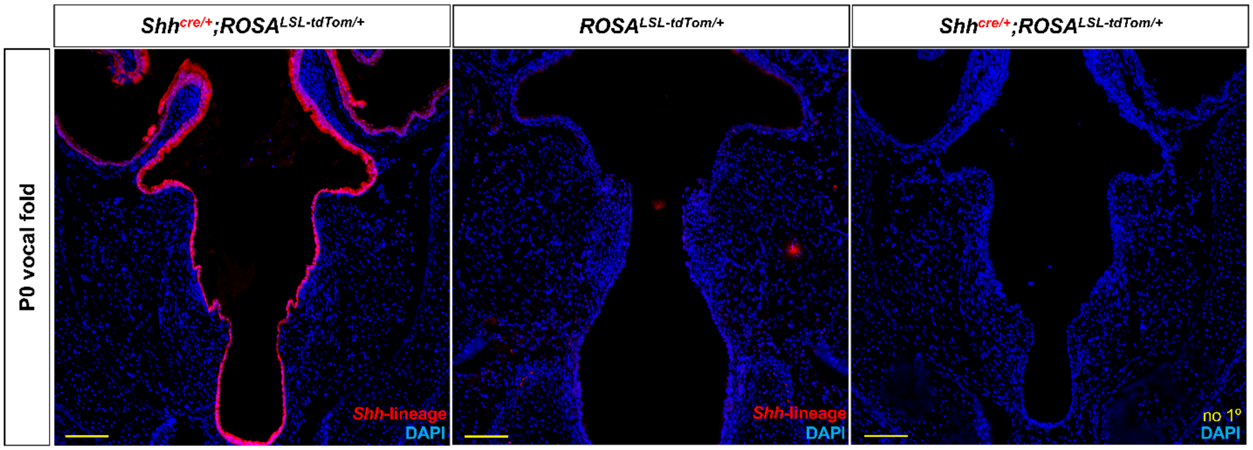

Fig. S5 Positive and negative tissue controls. IF analysis exhibited effective *tdTomato* recombination for cell-specificity to *shh* promoter using antiserum against red fluorescent protein (RFP) at P0 timepoint *(Shh$^{cre/+}$;ROSA$^{LSL\text{-}tdTom/+}$)*. Negative controls did not exhibit RFP expression when antiserum was applied, which suggests an effective Cre system for murine colonies *(ROSA$^{LSL\text{-}tdTom/+}$)* and when no primary antiserum was applied *(Shh$^{cre/+}$;ROSA$^{LSL\text{-}tdTom/+}$)*. Images taken at 20X magnification. Scale bar represents 100 µm for all images. WT: wildtype, RFP: red fluorescent protein, P0: postnatal day 0, shh: sonic hedgehog.

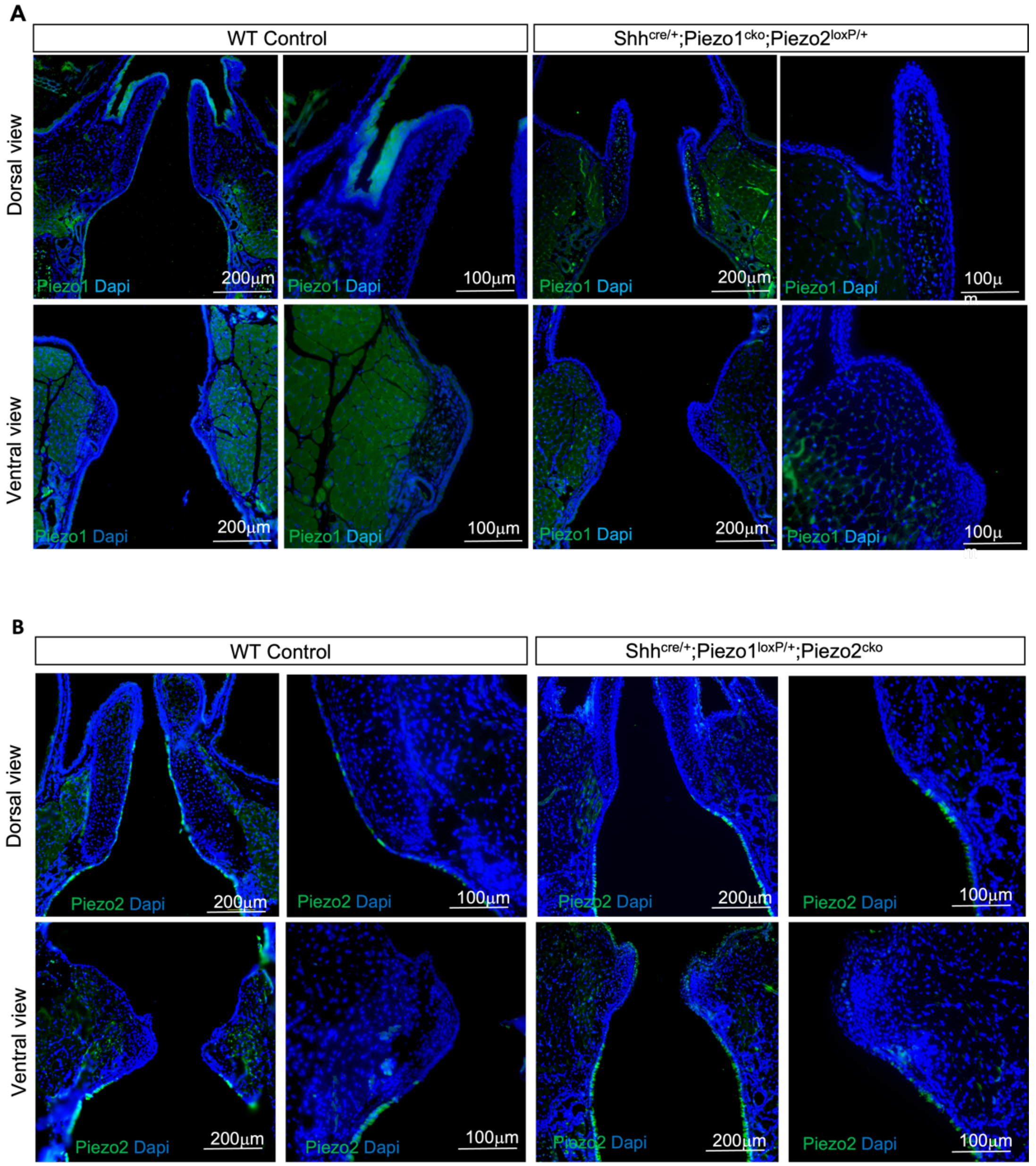

Fig. S6 (a) IF analysis of coronal sections for PIEZO1 (green) antibody exhibit no expression with antiserum following *Piezo1* genetic deletion compared to WT control epithelium. **(b)** IF analysis of coronal sections for PIEZO2 (green) antibody exhibit residual expression with antiserum following *Piezo2* genetic deletion compared to WT control epithelium. WT: wildtype.

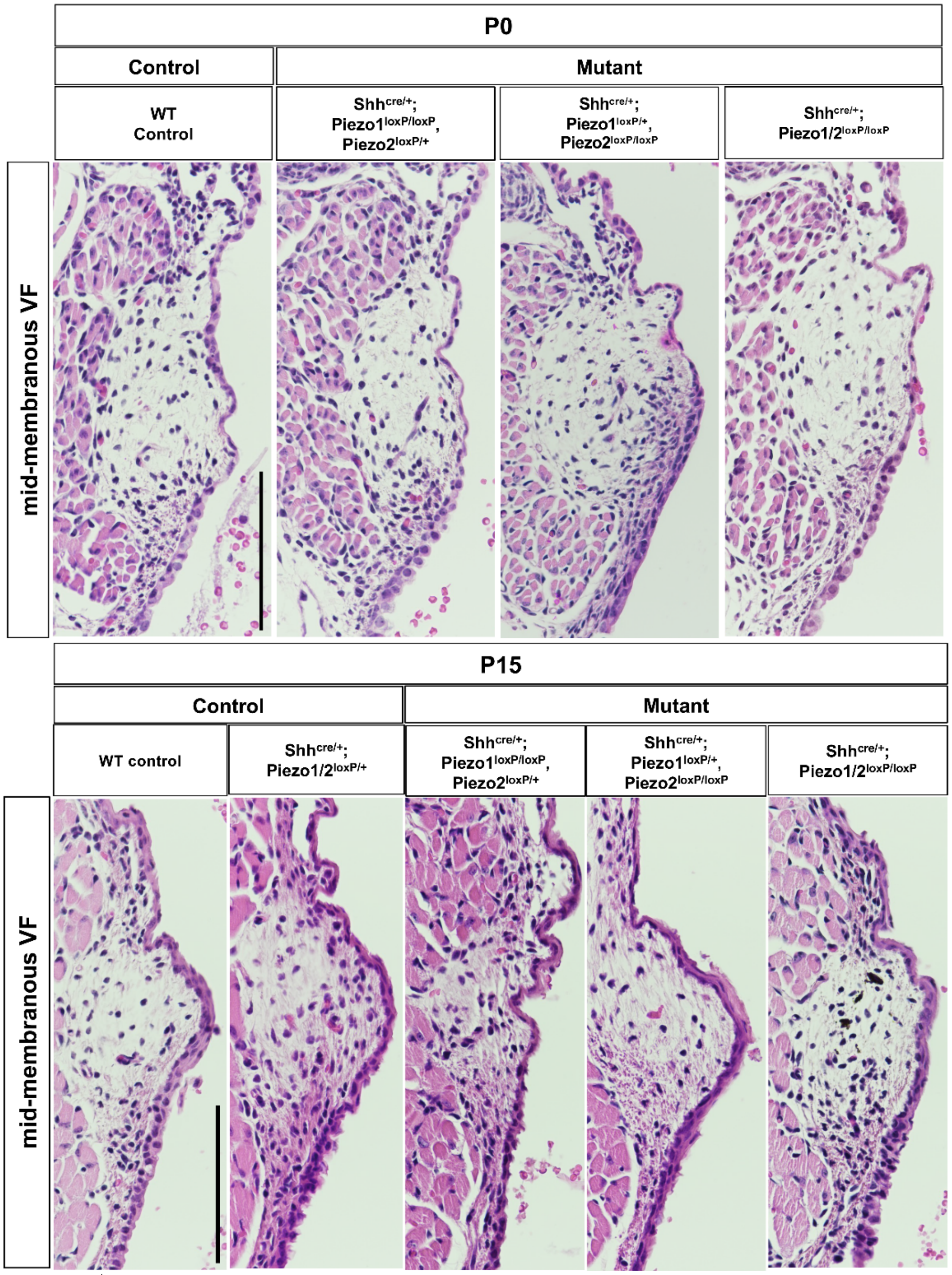

Fig. S7 *Shh$^{cre/+}$;Piezo1,Piezo2* single and compound mutant mice exhibit grossly normal vocal fold epithelium compared to heterozygote and WT controls at P0 and P15 timepoints. All images taken at 60X magnification. Scale bar represents 100 µm. WT: wildtype, VF: vocal fold, P: postnatal.

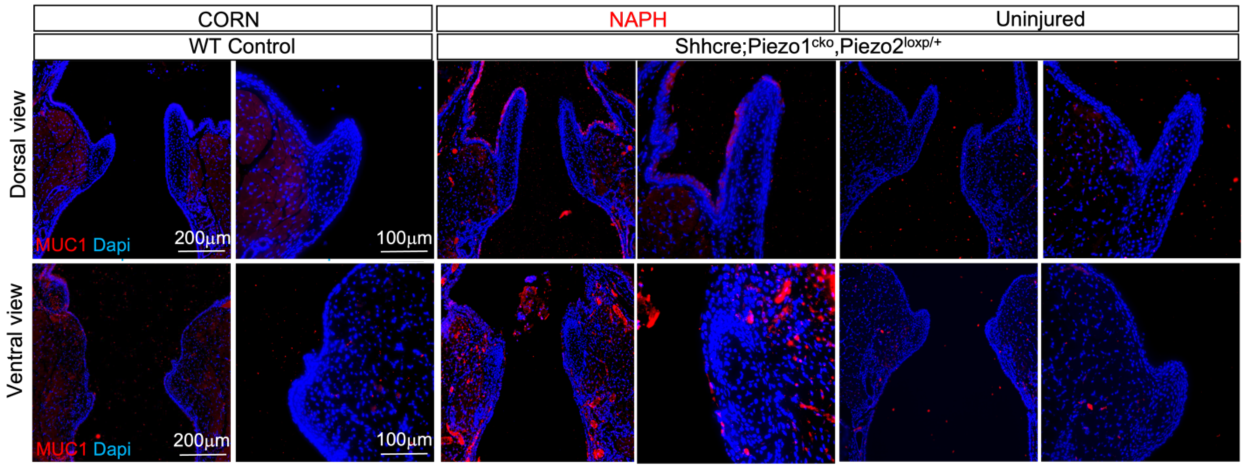

Fig. S8 20X and 30X magnification of WT control and *Shh*$^{cre/+}$;*Piezo1*^{cko},*Piezo2*$^{loxP/+}$ mutant epithelium with and without injury in coronal view exhibits upregulated MUC1 expression patterns to *Piezo1* mutant vocal fold epithelium and submucosal glands of the subglottis only following injury. DAPI is in blue. Scale bar represents 200 µm (20X) and 100 µm (30X).

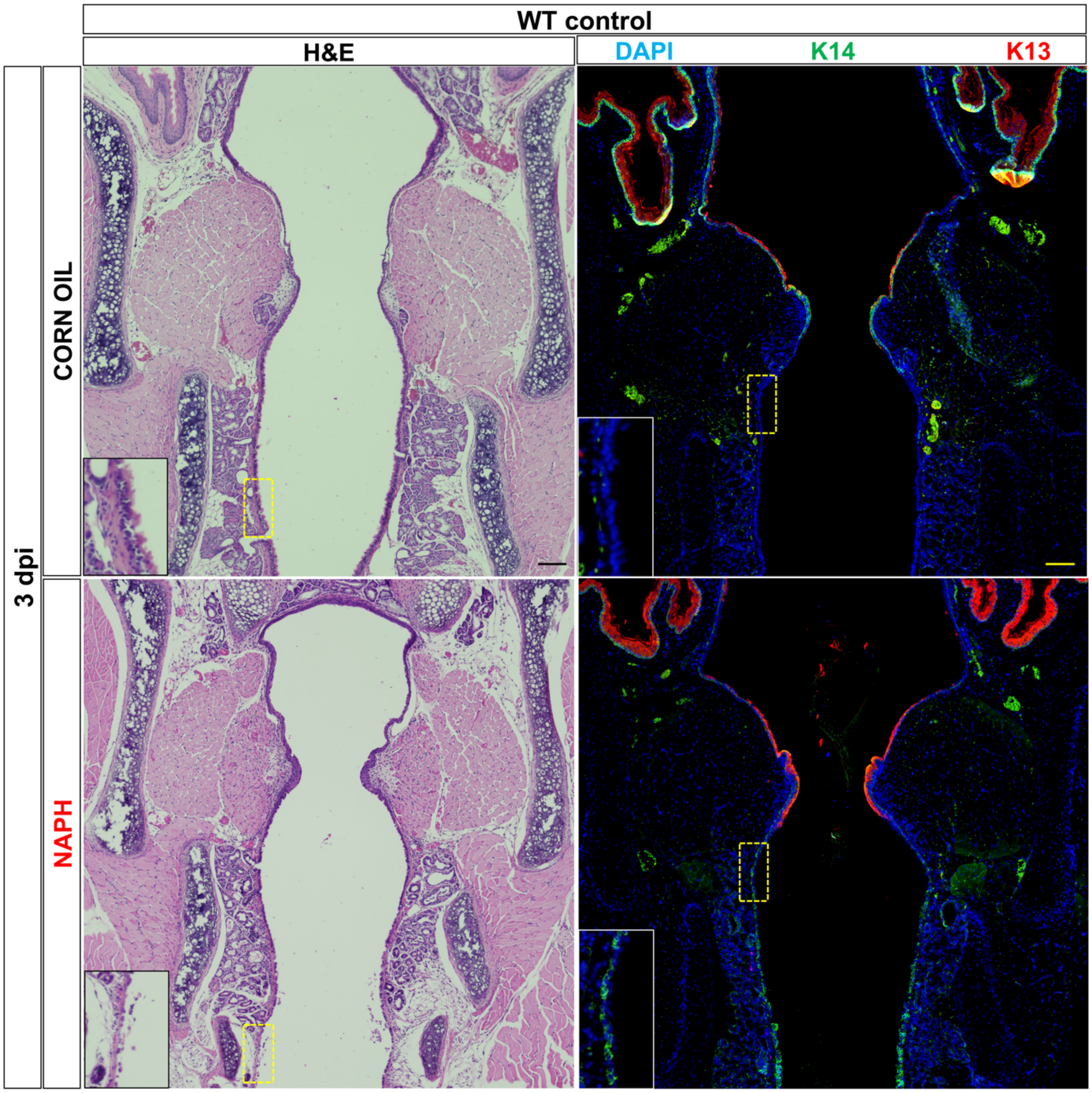

Fig. S9 Characterization of naphthalene injury model at 3dpi. H&E-stained slides exhibit hyperplasic changes to upper airway epithelium versus denuding to lower airway epithelium following NAPH injury. Yellow box indicates tracheal region with severe epithelial denuding. IF analysis revealed increased keratin (K) 14 (green) expression patterns along the entire airway epithelium with increased K13 (red) expression extending into the subglottic region following NAPH injury. DAPI is in blue. All images taken at 10X magnification. Scale bar represents 500 µm. WT: wildtype, dpi: days post injury, NAPH: naphthalene.

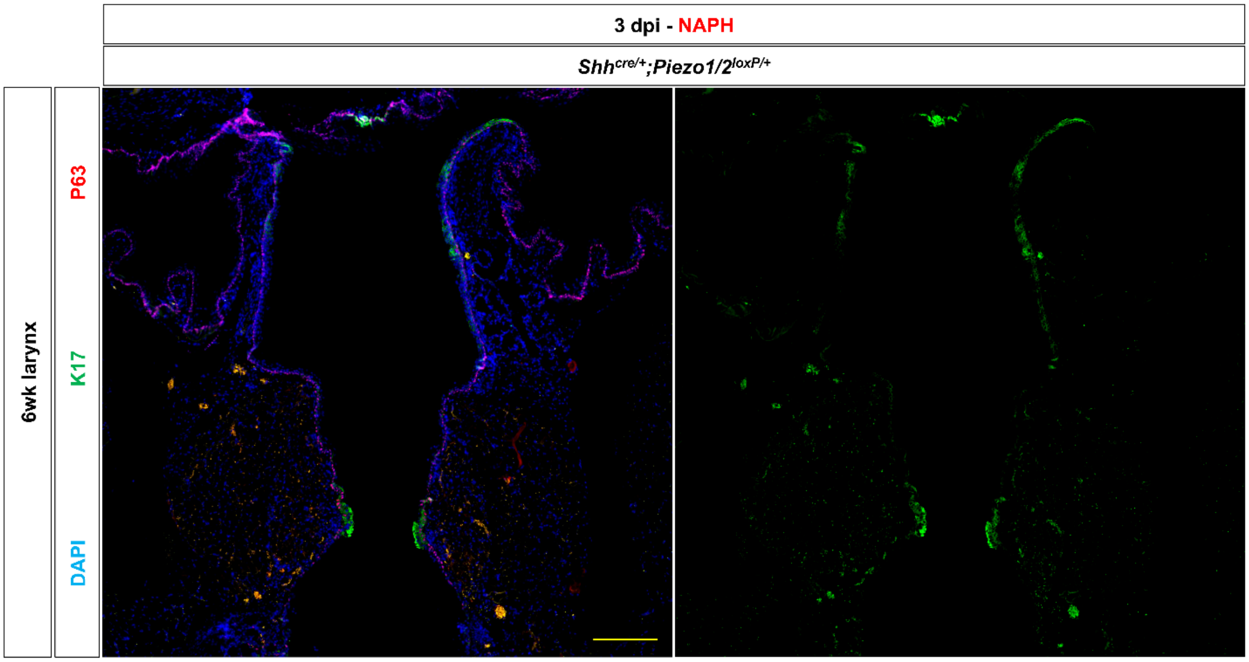

Fig. S10 10X magnification of *Shh*$^{cre/+}$;*Piezo1/2*$^{loxP/+}$ mutant larynx in coronal view exhibits preferential K17 expression patterns to vocal fold and laryngeal surface of epiglottis. DAPI is in blue. Image taken at 10X. Scale bar represents 500 µm. dpi: days post injury, NAPH: naphthalene , K: keratin.
